# RNAi‐suppression of barley caffeic acid *O*‐methyltransferase modifies lignin despite redundancy in the gene family

**DOI:** 10.1111/pbi.13001

**Published:** 2018-10-02

**Authors:** Paul Daly, Christopher McClellan, Marta Maluk, Helena Oakey, Catherine Lapierre, Robbie Waugh, Jennifer Stephens, David Marshall, Abdellah Barakate, Yukiko Tsuji, Geert Goeminne, Ruben Vanholme, Wout Boerjan, John Ralph, Claire Halpin

**Affiliations:** ^1^ Division of Plant Sciences School of Life Sciences University of Dundee at the James Hutton Institute Dundee UK; ^2^ Faculty of Sciences School of Agriculture, Food and Wine University of Adelaide Adelaide Australia; ^3^ UMR1318 INRA‐AgroParistech IJPB Universite Paris‐Saclay Versailles Cedex France; ^4^ Cell and Molecular Sciences James Hutton Institute Dundee UK; ^5^ Information and Computational Sciences James Hutton Institute Dundee UK; ^6^ Department of Biochemistry University of Wisconsin‐Madison Madison WI USA; ^7^ Department of Energy's Great Lakes Bioenergy Research Center The Wisconsin Energy Institute University of Wisconsin‐Madison Madison WI USA; ^8^ Department of Plant Biotechnology and Bioinformatics Ghent University Ghent Belgium; ^9^ VIB Center for Plant Systems Biology Ghent Belgium; ^10^ Present address: Fungal Physiology Westerdijk Fungal Biodiversity Institute and Fungal Molecular Physiology Utrecht University Utrecht The Netherlands

**Keywords:** *caffeic acid O‐methyltransferase* (COMT), lignin, brown‐midrib, barley (*Hordeum vulgare*), straw, Biofuels

## Abstract

*Caffeic acid O‐methyltransferase* (COMT), the lignin biosynthesis gene modified in many brown‐midrib high‐digestibility mutants of maize and sorghum, was targeted for downregulation in the small grain temperate cereal, barley (*Hordeum vulgare*), to improve straw properties. Phylogenetic and expression analyses identified the barley *COMT* orthologue(s) expressed in stems, defining a larger gene family than in brachypodium or rice with three *COMT* genes expressed in lignifying tissues. RNAi significantly reduced stem COMT protein and enzyme activity, and modestly reduced stem lignin content while dramatically changing lignin structure. Lignin syringyl‐to‐guaiacyl ratio was reduced by ~50%, the 5‐hydroxyguaiacyl (5‐OH‐G) unit incorporated into lignin at 10‐–15‐fold higher levels than normal, and the amount of *p*‐coumaric acid ester‐linked to cell walls was reduced by ~50%. No brown‐midrib phenotype was observed in any RNAi line despite significant COMT suppression and altered lignin. The novel *COMT* gene family structure in barley highlights the dynamic nature of grass genomes. Redundancy in barley COMTs may explain the absence of brown‐midrib mutants in barley and wheat. The barley COMT RNAi lines nevertheless have the potential to be exploited for bioenergy applications and as animal feed.

## Introduction

The properties of plant biomass are largely determined by its composition and in particular by the amount and structure of lignin. These properties influence the digestibility of crop biomass as animal feed (Gressel and Zilberstein, 2003) and its potential use as a renewable raw material for an emerging biorefinery industry producing biochemicals and biofuels (Gomez *et al*., 2008; Halpin *et al*., 2010; US‐DOE, 2006). The lignin content of plant biomass is negatively correlated with saccharification, the enzymatic release of simple sugars (Chen and Dixon, 2007; Van Acker *et al*., 2013), while changing the relative proportions of different lignin units is associated with changes to digestibility (Mechin *et al*., 2005) and saccharification after acid pretreatment (Studer *et al*., 2011; Van Acker *et al*., 2013). The possibility of optimising the content and structure of lignin in biomass to facilitate processes such as biofuel production is a very active area of current research worldwide.

In the C4 grasses maize (*Zea mays*) and sorghum (*Sorghum bicolor*), mutations in certain lignin biosynthesis genes, including *caffeic acid O‐methyltransferase* (*COMT*), give rise to a phenotype of brown midribs that is associated with lower lignin content and higher digestibility (Bout and Vermerris, 2003; Vignols *et al*., 1995). Such *bm* or *bmr* mutants are consequently marketed in the USA as superior forage and silage cultivars and some are reported to increase bioethanol yields (Dien *et al*., 2009). Most research has focussed on the maize *bm3 COMT* mutant which seems to have the greatest digestibility and feeding value improvement (Barrière *et al*., 2004). Although the lignin pathway is generally better characterised in dicots than monocots (Anterola and Lewis, 2002), COMT's main role in both types of plant appears to be to methylate 5‐hydroxyconiferaldehyde on the route to the synthesis of S units (Osakabe *et al*., 1999). Nevertheless, COMT is considered a multifunctional enzyme: in *Arabidopsis* it was shown to be involved in the biosynthesis of sinapate esters (Goujon *et al*., 2003), it has been annotated as a flavonol OMT (Muzac *et al*., 2000), and *Sorghum bicolor* COMT can methylate the flavones luteolin and selgin (Eudes *et al*., 2017).

The brown‐midrib phenotype has not been associated with *COMT* mutations in C3 grasses such as wheat (*Triticum* spp.) and barley (*Hordeum vulgare*), the dominant sources of straw biomass in temperate world regions. Substantial surplus wheat straw is available globally that could be used as a raw material for bioenergy (Copeland and Turley, 2008; Kim and Dale, 2004) but wheat is not a particularly tractable genetic system for research because of its large polyploid genome. In contrast, barley is an inbreeding true diploid for which substantial genetic and bioinformatic genomic resources are available (Hein *et al*., 2009; Mascher *et al*., 2017; Saisho and Takeda, 2011), and it is readily and efficiently transformed (Harwood *et al*., 2008). Barley is a particularly good model for polyploid wheat, diverging from a common ancestor only ~8–9 mya (Middleton *et al*., 2014). Apart from its use as a research model, barley is the fourth largest global cereal crop by production with ~144 million metric tonnes produced in 2014 (FAOSTAT, 2014). It is a staple food in countries such as Ethiopia, but in temperate regions is cultivated primarily for grain use for malting and animal feed (Slafer *et al*., 2002). The straw can also be used as fodder and forage but has potential for use as a raw material for biorefineries producing chemicals and second generation biofuels. Consequently, we aimed to downregulate *COMT* in barley to demonstrate the value for agriculture and industrial biotechnology of improving straw digestibility in the small grain temperate cereals.

## Results

### Identification of the *COMT* genes in barley

**Figure 1:**
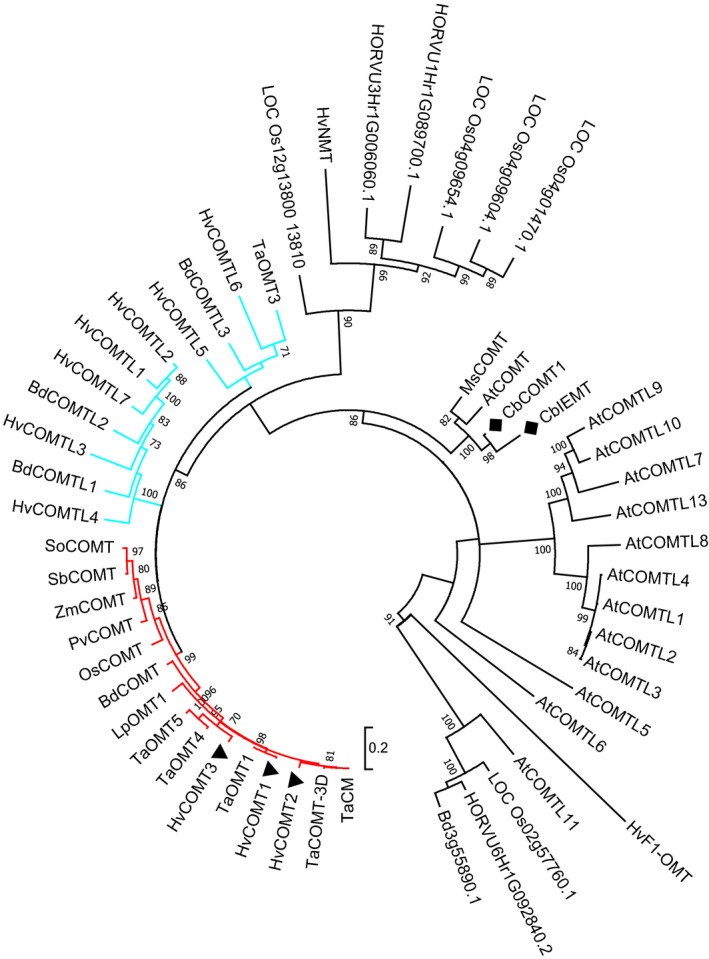
Maximum‐Likelihood unrooted phylogenetic tree with the *COMT* genes from barley, brachypodium, wheat, rice, perennial ryegrass, maize, alfalfa, sugarcane, switchgrass, *C. breweri* and *Arabidopsis* along with some *COMT‐like* genes and other genes that were returned in BLAST searches of barley, brachypodium and rice. For clarity, the clade containing the monocot *COMT* genes is highlighted in red and the clades containing the barley COMT‐likes in blue. The barley *COMT* genes are highlighted with solid black triangles. Bootstrapping values ≥70 from 100 trials are shown. The scale bar represents 0.2 amino acid substitutions per site. See Table S1 for the accession numbers and further information on the genes in the phylogenetic tree.

BLAST searches were performed in sequence databases for a phylogenetic analysis to identify *COMT* genes in barley. However, *COMT* genes cannot be identified by phylogeny alone; the closely related genes *CbCOMT1* and *CbIEMT* of *Clarkia breweri* (black diamond on phylogenetic tree, Figure 1), encode *O*‐methyltransferases with distinct substrate specificities, and only one is a COMT (Wang and Pichersky, 1999). Therefore, 13 conserved residues for COMT catalytic activity and binding/positioning of the substrates ferulic acid and 5‐hydroxyconiferaldehyde (Zubieta *et al*., 2002) were used along with phylogenetic analysis to identify COMT genes. This approach identified three *COMT* genes in barley and, notably, only one in brachypodium (Figure 1, red highlighted cluster of the tree). The encoded proteins all contained 12 out of 13 of the conserved residues (isoleucine I_316_ is substituted by a valine in several species) and the genes were annotated as *HvCOMT1*,* HvCOMT2*,* HvCOMT3* and *BdCOMT* (see Figure S2 for alignment). All four genes encoded a conserved Ser^123^ shown to be phosphorylated in poplar COMT, while only *HvCOMT2* encodes Ser^125^, an alternative phosphorylation site in poplar (Wang *et al*., 2015). In the phylogenetic analysis these *COMT* genes clustered closely with two well characterised monocot *COMT* genes: the maize *COMT* (*Zea mays*;* ZmCOMT*) which is knocked out in the *bm3* mutant due to insertions or deletions in the gene (Vignols *et al*., 1995) and the *COMT* gene from perennial ryegrass (*Lolium perenne*;* LpOMT1*) (Heath *et al*., 1998; Tu *et al*., 2010). Also in this clade was the single *COMT* gene in rice (*Oryza sativa*;* OsCOMT*) (Hamberger *et al*., 2007).

Several other genes from barley and brachypodium clustered closely with the monocot *COMT* genes clade (Figure 1, blue highlighted clusters) but all lacked some of the substrate binding/positioning residues and were therefore annotated as *COMT‐likes* (see Figure S2, Table S4). For example, *HvCOMTL1* (previously described by Sugimoto *et al*., 2003), *HvCOMTL2*,* HvCOMTL3*,* HvCOMTL4*,* HvCOMTL7*,* BdCOMTL1* and *BdCOMTL2* have an alanine (A_131_) substituted for the asparagine (N_131_) in *COMT* genes. Asparagine N_131_ is important for binding oxygenated propene side‐chains on lignin pathway intermediates whereas alanine A_131_ is important for non‐oxygenated propene side‐chains such as on eugenol (Louie *et al*., 2010; Wang and Pichersky, 1999). *HvCOMTL1*,* HvCOMTL2*,* HvCOMTL3*,* HvCOMTL4*,* HvCOMTL7*,* BdCOMTL1* and *BdCOMTL2* lack a catalytic histidine (H_269_) which functions in deprotonating the hydroxyl group. *HvCOMTL5*,* HvCOMTL6* and *BdCOMTL3* lack several of the conserved residues. *COMTL* genes are expected to have diverse substrates and functions distinct from those of the ‘true’ COMTs that function in lignin biosynthesis. This highlights the importance of incorporating an evaluation of COMT conserved residues in phylogenetic analysis in order to identify true COMTs that use ferulic acid and 5‐hydroxyconiferaldehyde substrates in lignin biosynthesis. Previous analyses of brachypodium genes based on homology alone identified four *COMT*s (Dalmais *et al*., 2013; Wu *et al*., 2013) but, of these, only Bd3g16530 is identified here as a true *COMT* and is denoted *BdCOMT* (*BdCOMT6* in Dalmais *et al*., and *BdCOMT4* in Wu *et al*.,) while the other genes, in our analysis, are *COMT‐likes* (*BdCOMTL1‐3*). Conversely, one of our barley COMTs, *HvCOMT2*, was previously suggested to be a flavone‐specific O‐methyltransferase (Zhou *et al*., 2008) but has all of the conserved residues of a functioning COMT and locates to the COMT clade.

Several rice *COMT‐like* genes (Hamberger *et al*., 2007) clustered in a separate clade along with three barley genes. One of these barley genes was reannotated as an N‐methyltransferase *HvNMT* involved in gramine biosynthesis by Larsson *et al*. (2006) from a previous erroneous annotation as a *COMT* gene (Lee *et al*., 1997), possibly suggesting that other genes in this clade might also be NMTs.

The three barley *COMT* genes (*HvCOMT1*(7H), *HvCOMT2*(3H) and *HvCOMT3*(6H)) are located on different chromosomes (see Table S1; Method S2). Barley chromosome 7H, where *HvCOMT1* is located, shares some synteny with the genomic location of *OsCOMT*,* BdCOMT* and *ZmCOMT* (Bennetzen and Chen, 2008; Vogel *et al*., 2010). *HvCOMT1, 2* and *3* are homologues of wheat *COMT* and *OMT* genes previously identified (Jung *et al*., 2008) (monocot COMT clade, Figure 1) and this is further supported by the shared synteny of the chromosome arms from wheat and barley that the genes mapped to (Table S5). *BdCOMTL1* and *BdCOMTL2* are a tandem duplication on chromosome 2 in brachypodium and *HvCOMTL1* and *HvCOMTL2* are on the syntenic barley chromosome 1H.

### Barley COMTs have different expression patterns

To investigate which COMT genes were expressed in barley stems, real‐time PCR was performed (delta‐delta Ct method) on the 2nd internode and the internode beneath the peduncle at different developmental stages. No expression was detected for *HvCOMT3* in these internodes. Expression of *HvCOMT1* and *HvCOMT2* were similar to each other in being higher in earlier compared to later internode stages, but the expression range of *HvCOMT2* was greater than *HvCOMT1* across the stages (Figure S5). In the internode beneath the peduncle when the spike was half to fully emerged, the expression of *HvCOMT2* was 100‐fold less than it was when the flag leaf was emerging. In contrast, there was only a tenfold difference in *HvCOMT1* expression across the same developmental stages. Expressed sequence tags (ESTs) in HarveEST#35 for *HvCOMT1* and *HvCOMT2* also come from a range of tissues while all ESTs for *HvCOMT3* are from roots (Table S6). Recent RNAseq data (Mascher *et al*., 2017) confirms *HvCOMT3* is predominantly expressed in roots and embryos while *HvCOMT1* and *HvCOMT2* are expressed in lignifying tissues including stems, roots, lemma, palea, and rachis, but to different levels. In the same dataset, none of the *HvCOMT‐likes* are expressed in stem tissue (Figure S6).

### Strategy to downregulate *COMT* genes in barley stems

The expression analyses indicated possible redundancy between *COMT* genes expressed in stems and therefore RNAi was chosen as the strategy to downregulate both *COMT* genes. A 634 bp fragment from *HvCOMT1* with 92% identity to *HvCOMT2* (and 90.4% identity to HvCOMT3) was used to form the inverted repeat sequences of the hairpin in the pIPKb007 RNAi vector under the control of the constitutive maize ubiquitin promoter. Regenerated plants were screened to identify those where *COMT* genes were downregulated.

To determine an appropriate tissue and developmental stage to screen, we investigated *O*‐methylation of caffeic acid in internodes at different developmental stages in the primary transformants (Figure S3a). Although caffeic acid can be *O*‐methylated by enzymes other than COMT, the assay reflects, at least in part, COMT enzyme activity *in planta*. *O*‐methylating activity varied with developmental stage with activity increasing, levelling off and then decreasing as internodes developed (Figure S3b). The second internode was chosen as the tissue to screen in plants 6 to 8 weeks after sowing, when activity is relatively high and stable (Figure S3b).

### COMT RNAi lines have reduced COMT activity

**Figure 2:**
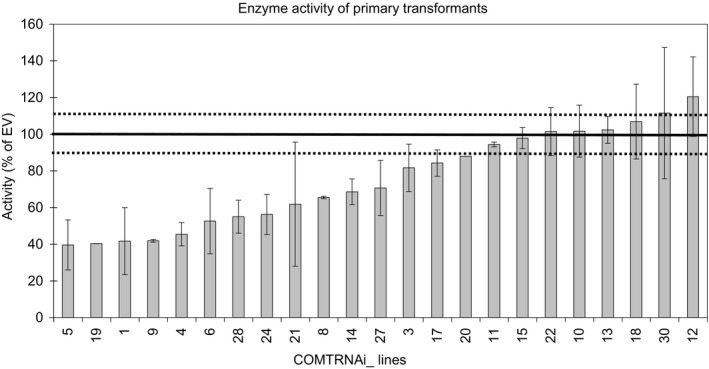
Summary graph of enzyme assay of the primary transformants. The lines on the graphs are shown in order of increasing enzyme activity. The thick black line at 100% indicates the activity of the EV controls from each run and the dotted lines are the average of the standard errors from the EV controls from the different runs. Between one and four stems were assayed from each line.

Twenty‐three independent primary transformants were assayed for reductions in COMT activity. Levels of biological variation differed between plants and this likely reflects slight differences in the developmental stage of replicate stems selected for assay. In several of the plants the activity was reduced to approximately 50% of the empty vector (EV) controls (Figure 2). In total, 12 lines were selected (11 lines with reduced activity and one line, COMTRNAi_26, which was not assayed). Southern analysis identified nine lines containing a single T‐DNA locus (Figure S4) and eight of these (COMTRNAi_1, 4, 5, 9, 14, 19, 26 and 28) were taken forward to the T1 generation for detailed analyses.

### COMT protein is substantially reduced in the COMT RNAi lines

**Figure 3:**
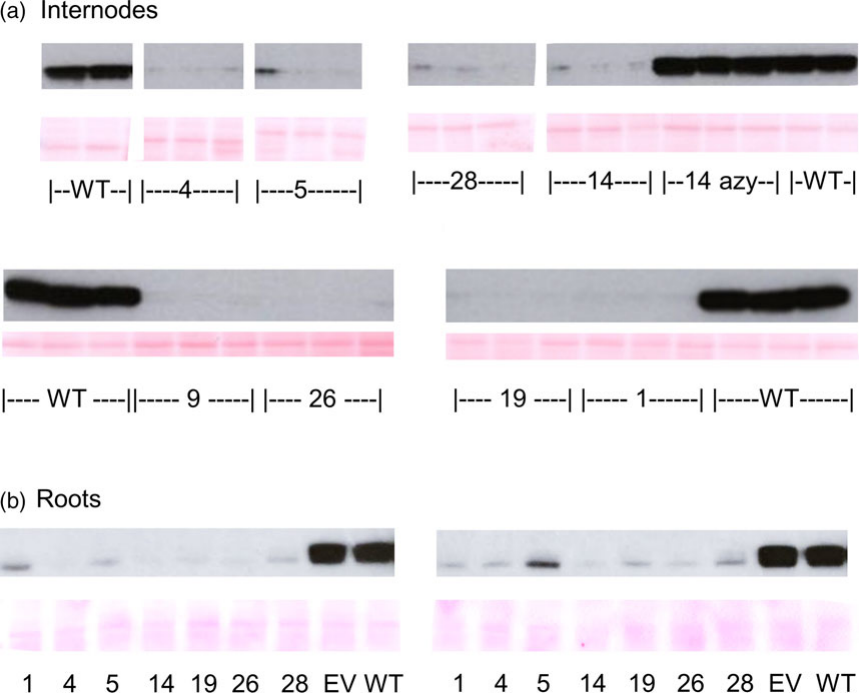
Western blot of crude protein extract from (a) internodes of the T1 lines and (b) roots of T3 lines probed with anti‐COMT antibodies. Wherever possible, crude extract from three homozygous plants was probed from each line along with three wild‐type and three azygous control plants (T1 plants that had lost the transgene due to segregation of the single T‐DNA locus). For the root samples, two plants were sampled from each of the lines at the tillering stage before stem elongation began. For COMTRNAi_1, 5, 26 and 28 one of the three plants was a hemizygote. Ponceau S staining is used to demonstrate equal protein loading.

To further characterise the lines, antibodies were raised against HvCOMT1 recombinant protein. Internodes from all lines showed substantial and similar reductions in COMT protein compared to the controls on western blots probed with the anti‐COMT antibodies (Figure 3a). Consistent with the fact that the RNAi was expressed from a constitutive promoter, COMT protein was also substantially reduced in roots (Figure 3b). The western blot along with the enzyme assay from the primary transformants showed that COMT activity and protein were reduced in the stems of the COMT lines.

### Expression of *HvCOMT1* and *HvCOMT2* is reduced in COMT RNAi stems

**Figure 4:**
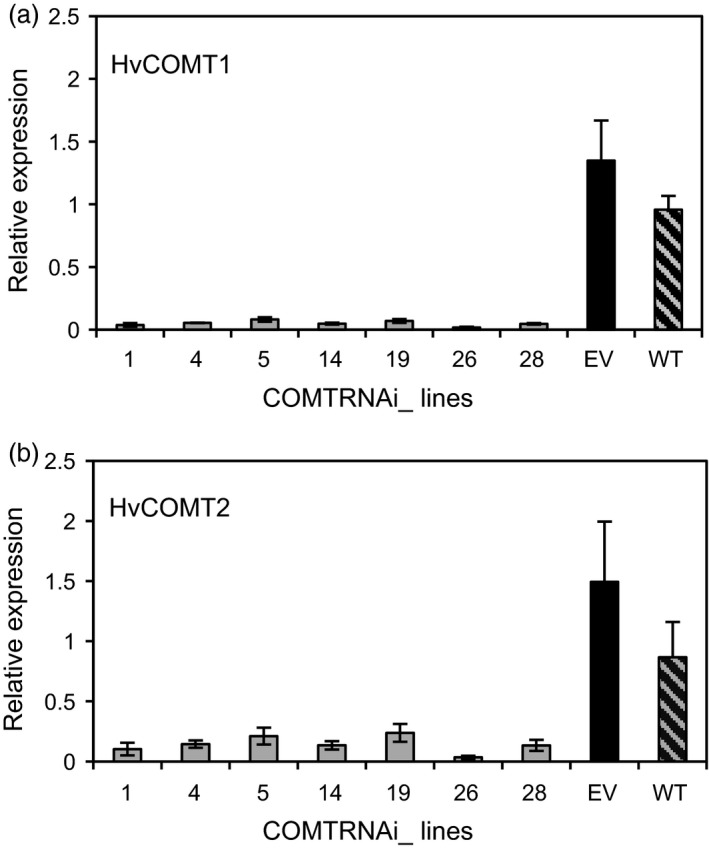
The expression of (a) *HvCOMT1* and (b) *HvCOMT2* in the 2nd internode when two nodes were present on the stem. The expression for each gene is relative to the expression of that gene in one of the wild‐type internodes. Three plants were sampled from each line and controls. The errors bars represent standard errors.

To investigate whether silencing of both *HvCOMT1* and *HvCOMT2* contributed to the reductions in COMT activity and protein levels, the second internode was sampled for real‐time PCR expression analysis when two nodes were present in the stem. The expression of both genes was reduced in the COMT lines compared to the controls with the expression of *HvCOMT1* reduced by 20‐80‐fold while the reduction in *HvCOMT2* expression was 5‐40‐fold (Figure 4a,b).

### Lignin structure is dramatically changed in COMT RNAi stems

**Figure 5:**
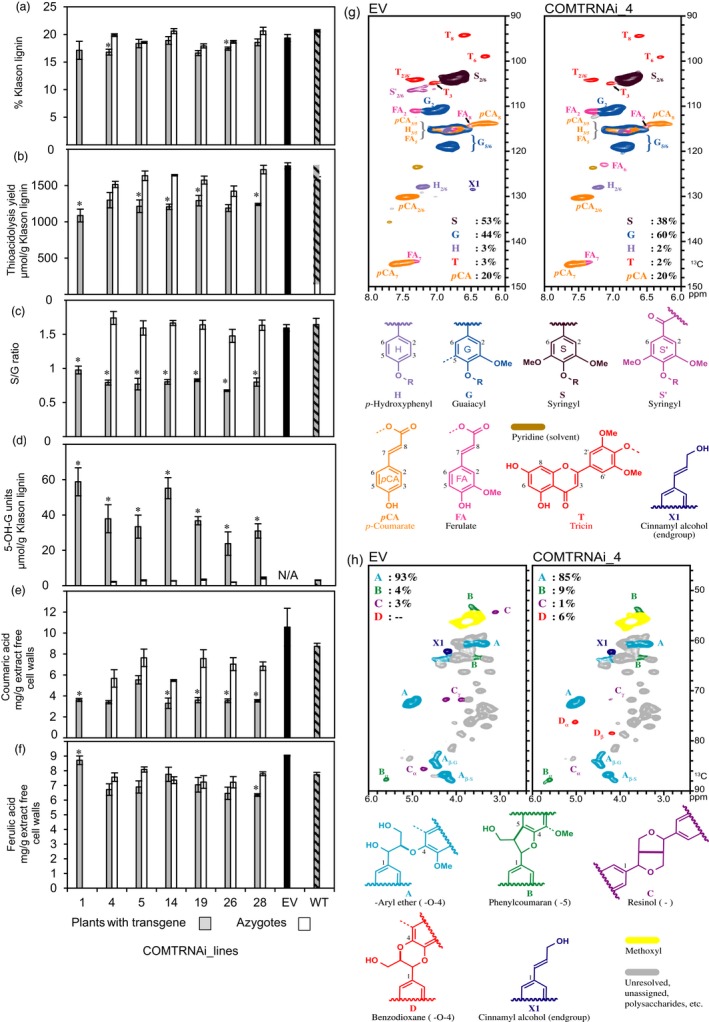
Analyses of extract‐free mature stems of T1 generation COMT RNAi lines and controls: (a) Klason lignin content, (b) thioacidolysis yield, (c) S/G ratio, (d) incorporation of the 5‐OH‐G unit, (e) thioacidolysis‐derived p‐coumaric acid derivatives (free acid and its EtSH addition product), (f) thioacidolysis‐derived ferulate derivatives (free acid and its EtSH addition product), (g) 2D NMR spectral sub‐plots of the major lignin subunits, and (h) the aliphatic region showing the major lignin units with their characteristic interunit bonds. Lines marked with a * are significantly different to the azygote controls or to the EV where no azygous controls were available (Student's *t*‐test *P* < 0.05). The error bars represent standard errors between biological triplicates. For COMTRNAi_1, 5, 26 and 28, one of the three plants was a hemizygote. NMR was performed on ‘enzyme lignins’ after cellulose treatment. ppm = parts per million. See Table S7 for further lignin data.

Extract‐free straw from the T1 COMT lines was subjected to detailed lignin analysis. Two lines, COMTRNAi_4 and 26, had Klason lignin contents significantly lowered by 15% and 7% compared to their respective azygous controls (*P *<* *0.05) (Figure 5a) while there was no significant difference in straw biomass (Figure S7). Lignin structure in the T1 COMT lines was evaluated by thioacidolysis. This analytical degradation specifically provides H, G and S thioethylated monomers from H, G and S lignin units only involved in labile β‐O‐4 bonds (Rolando *et al*., 1992), the major interunit bonds in native lignin. The yield of thioacidolysis products was significantly reduced in each of the lines by 20%–30% compared to the controls (*P *<* *0.05) (Figure 5b) and the S/G ratio was significantly reduced by approximately 50% (*P *<* *0.05) (Figure 5c). The reduction in S/G was accounted for by an approximate reduction of 30% in the proportion of S units in thioacidolysis products and a proportional increase of approximately 40% in G units (Table S7), consistent with COMT's main role in the methylation of 5‐hydroxyconiferaldehyde, a precursor of lignin S units. When COMT is downregulated, it is generally considered that the 5‐hydroxyconiferaldehyde substrate accumulates and is reduced by cinnamyl alcohol dehydrogenase (CAD) to form 5‐hydroxyconiferyl alcohol which is then incorporated into lignin as an unusual 5‐OH‐G unit. When subjected to thioacidolysis, the barley COMT lines released the 5‐OH‐G monomer at 10‐15‐fold higher levels compared to the wild‐type control (Figure 5d) (*P *<* *0.05). In addition to lignin‐derived monomers, thioacidolysis provided free *p*‐coumaric acid (*p*CA) and its EtSH addition product, both originating from *p*CA esters in the cell walls. Thioacidolysis yields of cell wall *p*CA from different grasses closely parallel the yields released by mild alkaline hydrolysis (Figure S8) demonstrating that they provide a true estimate of the amounts of *p*CA esters. In lignified grass cell walls, most *p*CA is ester‐linked to S lignin units (Ralph *et al*., 1994). In the COMT lines, in agreement with the reduction in S‐units, there was a significant reduction in the amount of *p*CA‐derived thioacidolysis compounds (Figure 5e) (*P *<* *0.05). By contrast to *p*CA units, COMT deficiency in barley did not systematically change the amount of ferulic acid (FA) and of its EtSH addition product released by thioacidolysis (Figure 5f), which suggests that cell wall‐linked FA units (ester‐ and/or ether‐linked) are not substantially affected. However, FA yields determined with thioacidolysis are an underestimate but are higher than estimates based on mild alkaline hydrolysis that breaks only the ester bonds.

NMR analysis was used to independently verify the major changes to lignin evident from thioacidolysis and to add further details. Barley lignin analysed by 2D NMR (Figure 5g,h) shows the typical dominance of G and S units (44% and 53% respectively) with minor contributions of H units (3%). As is typical of grass lignins, other aromatics are associated with the lignin component—*p*CA, an endunit on lignin side‐chains (20% on an S + G + H = 100% basis, but over‐represented due to its relaxation properties) and tricin (3%), a flavone relatively recently described as a component of monocot lignins (Lan *et al*., 2015, 2016). A preponderance of β‐aryl ether (β‐O‐4) units (93%) dominate with small contributions from phenylcoumaran (4%) and resinol (3%) units. In the COMT RNAi line, it is clear in the aromatic and double‐bond regions of the spectra, that S units are relatively reduced and G units are increased, and H‐units are essentially unchanged (Figure 5g). The spectra clearly show the benzodioxane structures (Figure 5h, structure D) that are diagnostically produced from the incorporation of the novel monolignol, 5‐hydroxyconiferyl alcohol, with these structures representing some 6% in the sidechain analysis, but being undetectable in the control line. The amount of tricin **T** was marginally reduced in the COMT line, dropping from 3% in the control to 2% in the COMT RNAi line. The level of *p*CA was apparently unchanged which is consistent with the thioacidolysis data's showing no significant reduction in *p*CA in this particular line (COMTRNAi_Line 4), although the levels of thioacidolysis‐released esterified cell wall *p*CA were reduced in other RNAi lines.

Extensive tissue sampling at various developmental stages in this work provided no evidence for differences in colour in the COMT RNAi lines compared to the controls in internodes, nodes, midribs, leaves or grains—even though the lignin content and structure was changed, no brown‐midrib or gold hull phenotypes were evident.

### Metabolite changes in COMT RNAi lines

In order to delve deeper into the consequences of COMT suppression at a molecular level, the bottom three internodes from two COMT RNAi lines were subjected to both transcript and metabolite profiling along with control lines. Internode phenolic metabolites were extracted and analysed via UHPLC‐MS. Approximately 4924 profiled compounds had an abundance above 100 counts in at least one sample. Compounds (*m/z* traces) were selected for further consideration if their abundance was significantly (*P* < 0.01) different in both COMT RNAi lines compared to controls, showing at least a threefold change and an average abundance of ≥100 counts in either plant group. This generated a list of 130 *m/z* traces with a higher intensity in the COMT RNAi lines and six *m/z* traces with a lower intensity (Table S8). The 130 higher intensity *m/z* traces could be assigned to 108 compounds (some compounds give rise to more than one *m/z* trace). Based on accurate *m/z*, retention time and MS/MS fragmentation, we could characterize the structure of nine of the 108 compounds (Table 1, Figure S9). Four 5‐hydroxyconiferyl alcohol‐containing oligolignols were found to accumulate in the COMT RNAi lines; G(8‐O‐4)5‐OH‐G (compound **1**), S(8‐O‐4)5‐OH‐G (compound **2**), and two isomers of G(8‐O‐4)S(8‐O‐4)5‐OH‐G (compound **3** and **4**). However, the *m/z* with the highest intensity was assigned to 5‐hydroxyconiferyl alcohol linked to a hexose moiety (5‐hydroxyconiferyl alcohol + hexose 1, compound **5**). In addition, two other 5‐hydroxyconiferyl alcohol conjugates could be structurally resolved: 5‐hydroxyconiferyl alcohol + hexose 2 (compound **6**) and 5‐hydroxyconiferyl alcohol + acetylhexose (compound **7**). Also two caffeyl alcohol conjugates were found to accumulate in the COMT RNAi lines: caffeyl alcohol + hexose (compound **8**) and caffeyl alcohol + acetylhexose (compound **9**).

**Table 1:** List of structurally characterized compounds with a different abundance in the internodes of COMT RNAi lines as compared to controls

Number	tR	*m/z* experimental	Name	*m/z* theoretical	Δppm	COMTRNAi_14	COMTRNAi_4	EV	WT
						Mean ± S.E.M.	Mean ± S.E.M.	Mean ± S.E.M.	Mean ± S.E.M.
Compounds with increased abundance in COMT RNAi lines									
1	13.10	209.0804	S(8‐O‐4)5‐OH‐G^a^	209.0819	−7.10	675 ± 354	120 ± 103	b.d.l.	b.d.l.
2	13.36	179.0692	G(8‐O‐4)5‐OH‐G^a^	179.0714	−12.30	493 ± 199	110 ± 103	b.d.l.	b.d.l.
3	14.86	599.2137	G(8‐O‐4)S(8‐O‐4)5‐OH‐G 1	599.2134	0.60	15 092 ± 4356	3374 ± 2229	b.d.l.	b.d.l.
4	15.55	599.2123	G(8‐O‐4)S(8‐O‐4)5‐OH‐G 2	599.2134	−1.80	4291 ± 1238	920 ± 759	b.d.l.	b.d.l.
5	3.73	357.1227	5‐hydroxyconiferyl alcohol + hexose 1	357.1191	10.10	234 359 ± 49 825	69 993 ± 38 025	1107 ± 311	1096 ± 780
6	2.76	357.1194	5‐hydroxyconiferyl alcohol + hexose 2	357.1191	0.80	947 ± 127	437 ± 160	b.d.l.	b.d.l.
7	5.83	399.1301	5‐hydroxyconiferyl alcohol + acetyl hexose	399.1297	0.90	4843 ± 1186	1334 ± 668	b.d.l.	b.d.l.
8	3.69	327.1087	Caffeyl alcohol + hexose	327.1085	0.80	10 753 ± 790	4888 ± 1876	1006 ± 397	1037 ± 521
9	5.91	369.118	Caffeyl alcohol + acetyl hexose	369.1191	−3.10	2428 ± 355	901 ± 331	b.d.l.	b.d.l.
Compounds with reduced abundance in COMT RNAi lines									
10	12.53	433.1504	Sox(8‐O‐4)S	433.1504	0.00	1715 ± 1116	292 ± 382	3081 ± 2671	4395 ± 5849
11	14.48	659.2330	S(8‐O‐4)Sox(8‐O‐4)S	659.2346	−2.50	101 ± 135	b.d.l.	896 ± 1142	1080 ± 1686

a Compounds detected as in‐source fragments as described in Figure S9. Images of these structurally characterised compounds listed above are included in Figure S9. *t*_R_: retention time, Δppm: mass difference between *m/z*_experimental_ and *m/z*_theoretical_ in parts per million, S.E.M.: standard error of the mean, b.d.l.: below detection limit (set at 100 counts). For full method see Method S1.

The six *m/z* traces with a lower intensity in COMT lines originated from six different compounds, two of which could be structurally characterized (Table 1, Figure S9). Both were oligolignols which contain only S subunits: Sox(8‐O‐4)S (**9**) and S(8‐O‐4)Sox(8‐O‐4)S.

### Transcriptome changes in COMT RNAi lines

To evaluate the effect of *COMT* downregulation on gene expression in internodes, transcript profiling was performed on two COMT RNAi lines and control lines. Genes that were significantly (*P* < 0.01) differentially regulated in both COMT RNAi lines compared to controls were filtered for those showing at least a threefold change. Only four genes were substantially up‐regulated in COMT RNAi lines according to these criteria (Table S9); a lectin‐like receptor protein kinase, a protein of unknown function (and questionable gene model), an F‐box protein, and a methyl esterase. There were 14 genes significantly down‐regulated in COMT RNAi plants; *HvCOMT1* itself was most reduced by 24‐fold compared to controls. Other down‐regulated genes included a galactan synthase, *HORVU6Hr1G092840.2* encoding an OMT enzyme with unknown substrate, two zinc finger transcription factors, a F‐box protein, a kinase regulator and a cyclin (Table S9). *HvCOMT2* expression was reduced by 13‐fold and 17‐fold in the two COMT RNAi lines and would have been the second most greatly repressed gene but missed our stringent significance threshold due to variation within the controls (*P* values of 0.13 and 0.07). As anticipated from our earlier expression analysis, expression of *HvCOMT3* was not detected in control or RNAi internodes.

### Saccharification of some COMT RNAi lines is increased

**Figure 6:**
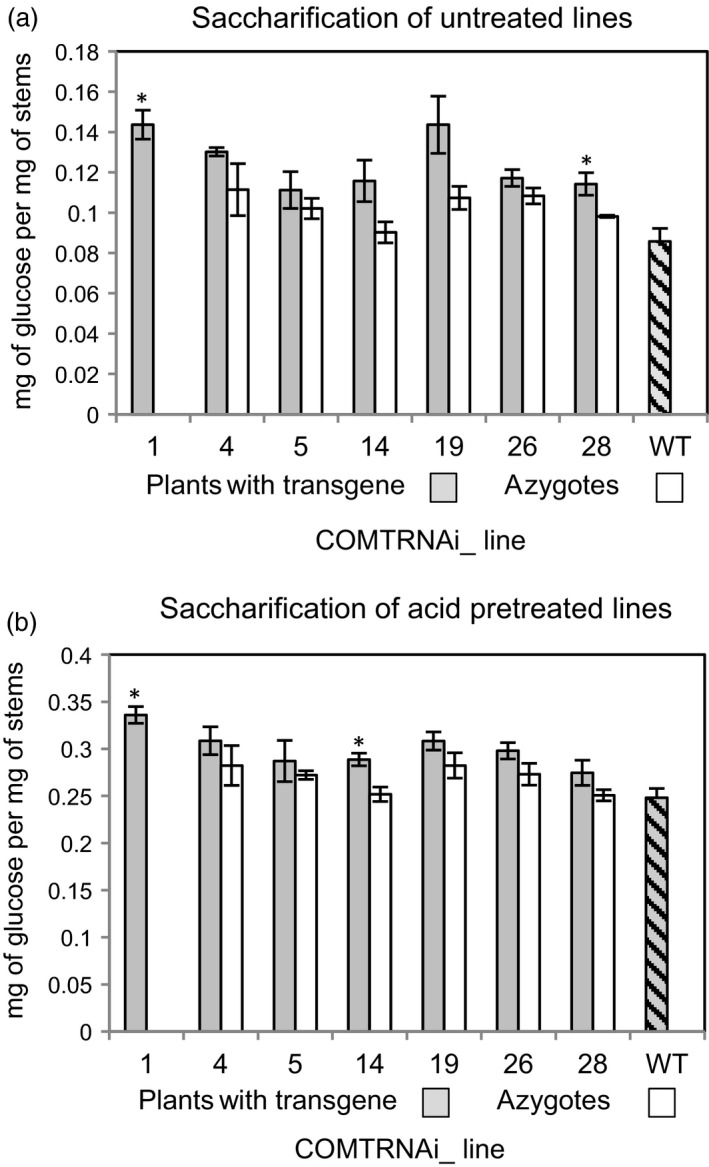
Saccharification of the COMT RNAi lines and controls from the T1 generation (a) without a pretreatment and (b) after an acid pretreatment. Lines marked with a * are significantly different to the azygous controls or to the wild‐type where no azygous controls were available (Student's *t*‐test, *P* < 0.05).

Straw from the COMT RNAi lines was subjected to saccharification without a pretreatment and after an acid pretreatment (Figure 6a,b). All RNAi lines showed a promising and consistent trend of higher saccharification compared to their corresponding azygote lines and wild‐type, but the difference was only statistically significant for lines COMTRNAi_1 and 28 without a pretreatment, and for lines COMTRNAi_1 and 14 after the acid pretreatment, representing approximately 16%–20% improvements in sugar release.

## Discussion

We show here that barley has a larger *COMT* gene family than brachypodium or rice suggesting *COMT* duplication in the barley lineage since its evolution from a common ancestor. This is consistent with the extensive gene duplication and expansion of specific gene families revealed in the barley reference sequence (Mascher *et al*., 2017). All three barley COMTs retain the amino acid residues essential to COMT activity and are preferentially expressed in lignifying tissues strongly suggesting that all three functions in lignin biosynthesis. Nevertheless, duplication seems to have been followed by some divergence in expression pattern, possibly reflecting subfunctionalization in different tissues or cell types (Ober, 2010). Several *COMT*s previously identified in wheat (Jung *et al*., 2008; Ma and Xu, 2008; Wang *et al*., 2018) are homologues of the barley *COMT* genes. *COMT* duplication events have also been noted in ryegrass (*Lolium perenne*) (van Parijs *et al*., 2015).

Given the redundancy in barley *COMT* genes, RNAi was an appropriate silencing strategy and was effective in suppressing both *HvCOMT1* and *HvCOMT2*. Reductions in enzyme activity in the primary transformants were relatively moderate compared to reductions in *HvCOMT* expression and protein levels. This may reflect greater specificity of the antibodies compared to the enzyme assay where other O‐methyltransferases might contribute background activity. Similarly in the maize *bm3* mutant, anti‐COMT antibodies could not detect residual COMT protein but enzyme activity was merely reduced (Piquemal *et al*., 2002). Nevertheless, expression of *HvCOMT1* and *HvCOMT2* is not abolished in our barley RNAi lines, COMT protein and activity are still present, albeit greatly reduced to levels sufficient to cause significant changes to lignin content and structure.

Lignin content was reduced in two barley COMT RNAi lines by 10%–15%. This compares to reductions in Klason lignin content of 25% and 28% when COMT was suppressed in maize (Piquemal *et al*., 2002) and to reductions of up to 16% of acetyl bromide lignin when COMT was suppressed in perennial ryegrass (Tu *et al*., 2010). Comparisons are complicated, however, because lignin content was measured at different developmental stages and by different methods in each study. Reduced thioacidolysis yields in the COMT RNAi lines are an indication of changes to lignin structure with a greater proportion of resistant bonds in the lignin. Reductions in the S/G ratio of ~50% in the barley RNAi lines were less than that in knock‐out mutants in maize and Arabidopsis where S units were reduced by ~70% (Barrière *et al*., 2004) or more (Goujon *et al*., 2003), respectively. The level of incorporation of the 5‐OH‐G unit was similar to that measured in the maize *bm3* mutant (Barrière *et al*., 2004), maize antisense RNA transgenic lines (Piquemal *et al*., 2002) and brachypodium mutants (Dalmais *et al*., 2013; Ho‐Yue‐Kuang *et al*., 2016) and higher than that measured in the *Arabidopsis* mutant (Goujon *et al*., 2003). To our knowledge, this is the first reported quantification of the 5‐OH‐G unit in a temperate cereal. The lack of a consistent reduction in thioacidolysis‐released ferulic acid is similar to what was found in COMT down‐regulated maize antisense RNA lines where there was even a slight increase in ferulic acid released by mild alkaline hydrolysis (Piquemal *et al*., 2002). Recently, a new lignin sub‐unit, tricin, has been described in grasses (Lan *et al*., 2015) and COMT has been implicated in its biosynthesis (Eudes *et al*., 2017; Fornalé *et al*., 2017). Barley appears to have only low levels of tricin compared to some other *Pooideae* (e.g. oats, wheat and brachypodium), with just 0.65 mg/g cell wall compared to 7.15 mg/g for oats (Lan *et al*., 2016). In this study, we detected a reduction to 2% of tricin in barley cell walls after COMT suppression, but levels in control plants were only modestly higher at 3%. In sorghum, similar 2D NMR spectroscopy of *bmr12 COMT* mutant biomass showed that it also had only 2% of tricin in cell walls, but levels in wild‐type sorghum were higher at 5% (Eudes *et al*., 2017). Nevertheless our data are consistent with the proposal that COMT is involved in the synthesis of both S lignin units and tricin (Eudes *et al*., 2017).

The maintenance of basal levels of *HvCOMT1* and *HvCOMT2* expression in the RNAi stems may explain the moderate level of other transcriptional changes. Given this, the number of metabolites that show altered abundance in the RNAi plants is perhaps surprising. Two less abundant metabolites were identified as α‐oxidized β‐O‐4‐ether oligomers of sinapyl alcohol (Sox(8‐O‐4)S, compound **10**; and S(8‐O‐4)Sox(8‐O‐4)S, compound **11**) (Figure S9). A reduction in the production of sinapyl alcohol in the RNAi plants is consistent with the reduction in S lignin and both result from the deficiency in COMT‐mediated conversion of 5‐hydroxyconiferaldehyde to sinapaldehyde, the precursor of sinapyl alcohol. The structure of Sox(8‐O‐4)S could be proven by an authentic standard (Tsuji *et al*., 2015), but has not yet been described in plants. The origin of the oxidation of the α‐position of β‐O‐4‐ethers is currently unknown, but has been observed in wild‐type Arabidopsis in 8‐O‐4‐dimers of coniferyl alcohol with either a second coniferyl alcohol (as in Gox(8‐O‐4)G) or ferulic acid (as in Gox(8‐O‐4)ferulic acid; Mnich *et al*., 2017; Tsuji *et al*., 2015).

The majority of the 108 compounds that were increased in the COMT RNAi lines are of unknown identity. Those containing 5‐hydroxyconiferyl alcohol (compound **1**–**7**) likely originate from the overproduction of the COMT substrate, 5‐hydroxyconiferaldehyde. This can be converted to coniferyl alcohol, presumably via CAD activity, and incorporated into benzodioxane oligolignols (compound **1**–**4**) and the benzodioxane structures in the lignin of COMT RNAi plants. Benzodioxane oligolignols have also been found in COMT‐deficient poplar and Arabidopsis (Morreel *et al*., 2004; Vanholme *et al*., 2010, 2012a,b). Not all 5‐hydroxyconiferyl alcohol may be used for lignification, however. Hexose and acetylhexose conjugates of 5‐hydroxyconiferyl alcohol (compound **5**–**6** and **7,** respectively) also accumulate in COMT RNAi plants and may be destined for vacuolar storage (Dima *et al*., 2015).

A striking observation is the accumulation in COMT RNAi plants of caffeyl alcohol conjugated to hexose (compound **8**) or acetyl hexose (compound **9**). This suggests that either caffeyl alcohol or caffealdehyde serve as a substrate for HvCOMT1, HvCOMT2 or both. Caffealdehyde has long been considered as an intermediate of the lignin pathway in several plant species (reviewed in Boerjan *et al*., 2003). A biosynthetic route to coniferaldehyde of caffeoyl‐CoA → caffealdehyde → coniferaldehyde, catalysed by CCR and COMT, would bypass the more commonly described route caffeoyl‐CoA → feruloyl‐CoA → coniferaldehyde, catalysed by CCoAOMT and CCR. This bypass‐route has been shown to be present in alfalfa (Lee *et al*., 2011; Parvathi *et al*., 2001; Zhou *et al*., 2010). Caffeyl alcohol has also been found as a monomer in lignin of CCoAOMT downregulated *Pinus radiata* (Wagner *et al*., 2011), in seeds of vanilla and in several cacti (Chen *et al*., 2012). However, our data are the first *in‐planta* evidence that the bypass‐route via caffealdehyde also occurs in grasses.

The changes described in lignin content and structure in COMT RNAi plants are likely to be beneficial for saccharification and digestibility, and moderate increases to saccharification were measured in some lines. Reduced lignin content is generally correlated with improvements in saccharification (Chen and Dixon, 2007) and downregulation or mutation of *COMT* has increased saccharification and/or biofuel production in switchgrass and sorghum (Dien *et al*., 2009; Fu *et al*., 2011; Saballos *et al*., 2008; Van Acker *et al*., 2013). The effect of the proportion of S units in lignin on digestibility is controversial; one study claims that the structure of lignin does not affect fermentation by ruminant microflora (Grabber *et al*., 2009) while another found an inverse correlation between digestibility and S lignin content (Mechin *et al*., 2005). Effects on saccharification are likely to depend on the pretreatment used, as reported by Studer *et al*. (2011). Incorporation of 5‐OH‐G units into lignin has been hypothesised as beneficial for saccharification; the quinone methide that forms during monomer coupling can be internally trapped by the ‐OH group on a 5‐OH‐G unit in lignin forming benzodioxane units instead of linking to polysaccharides, and that reduction in cross‐linking is likely to improve the access for saccharifying enzymes (Ralph *et al*., 2004; Vanholme *et al*., 2012a,b).

*COMT* duplication events in barley and wheat are sufficient to explain why no *brown‐midrib* or *gold‐hull* mutants associated with *COMT* have been identified in these small grain temperate cereals. We have evidence that *orange lemma* mutants are the barley equivalent of maize *brown‐midrib* and rice *gold‐hull* but none of the *orange lemma* mutants we have characterised are mutants in COMT (Stephens J, Reetoo N, Daly P, Waugh R, Druka A, Lapierre C and Halpin C, unpublished). Contrary to previous reports (Dalmais *et al*., 2013; Wu *et al*., 2013), our phylogenetic analysis identified a single true COMT gene in brachypodium, suggesting that brown‐midrib phenotypes might emerge if *COMT* was fully knocked out in this species. Various hypotheses were proposed to explain why brown‐midrib phenotypes had not been seen in C3 grasses, but brachypodium plants with brown midribs (or brown‐red lignified tissues) were recently described; all were plants severely suppressed or mutated in CAD (Trabucco *et al*., 2013; d'Yvoire *et al*., 2013). The existence of brachypodium plants sufficiently deficient in COMT to be expected to develop brown‐midrib phenotypes has not been definitively evidenced. A mutant in the brachypodium lignin COMT has been identified but displays only moderately altered lignification and the mutant enzyme is still functional (Ho‐Yue‐Kuang *et al*., 2016). Similarly, transgenic plants overexpressing artificial microRNA designed to silence brachypodium COMT did not have significant changes to S lignin (Trabucco *et al*., 2013) suggesting that they were not sufficiently COMT‐suppressed. Consequently, it is likely that a full knock‐out of COMT in brachypodium (or other species) will be necessary before brown‐midrib phenotypes are seen or their absence can reasonably prompt other explanations. In this context, it is interesting that COMT is reported to be the third most abundantly expressed gene in poplar stem‐differentiating xylem, accounting for 6% of the proteome (Lin *et al*., 2013; Shuford *et al*., 2012) and its near absence is thought necessary before S lignin content is reduced (Wang *et al*., 2014). In barley and wheat, the difficulties in effectively silencing gene activity to near abolition are likely to be exacerbated when more than one *COMT* gene needs to be suppressed. For example, our microarray data comparing the COMT RNAi lines with controls showed that, despite efficient gene downregulation, *HvCOMT1* and *HvCOMT2* expression could still be detected at 4% and 6%–7% of control plant values, respectively.

The ability to modify lignin differentially in specific tissues would also have great value in lignin engineering, for example enabling the production of crops that have more digestible stems (less lignin) and roots that sequester more carbon in soil (more lignin). The kind of gene duplication and expansion events described here for barley COMTs could in some cases enable such tissue specific manipulation, if gene sequences and expression patterns have diverged sufficiently to allow individual genes expressed in specific tissues to be targeted for suppression by RNAi. Tissue specific promoters might also place appropriate limitations on RNAi expression, albeit with the complication that small silencing RNAs might move between tissues.

The advent of CRISPR‐mediated targeted gene manipulation in plants offers real possibilities for more precise and effective gene manipulations. By careful selection of guide RNA sequences, several homologous genes (multiple gene family members, such as *HvCOMT1* and *HvCOMT2*, or homeologous genes in polyploid species) can be targeted for mutation while other closely related genes are avoided. Knock out of multiple COMT genes/homeologues in stems of barley and wheat might provide improved cereal straw for use as animal feed or as a feedstock for industrial processing in temperate regions of the world.

## Materials and methods

### Sequence retrieval and phylogenetic analysis

Barley, brachypodium and rice sequences with >40% identity to maize *ZmCOMT* (M73235) (Collazo *et al*., 1992) were retrieved from sequence databases and used for phylogenetic analysis along with published COMT genes from perennial ryegrass (Heath *et al*., 1998), sorghum (Bout and Vermerris, 2003), alfalfa (Zubieta *et al*., 2002), switchgrass (Fu *et al*., 2011), sugarcane (Jung *et al*., 2012; Selman‐Housein *et al*., 1999), arabidopsis *COMT* and *COMT‐like* genes from (Raes *et al*., 2003), several *wheat* COMT or OMT genes (Jung *et al*., 2008; Ma and Xu, 2008; Wang *et al*., 2018), and two *Clarkia brewer* genes (Wang and Pichersky, 1999). After importing aligned sequences into MEGA7, a maximum likelihood (ML) tree was constructed with JTT (Jones *et al*., 1992) + G + I as the model with five discrete gamma categories. All sites from the Gblocks‐selected subset of the alignment (Figure S1) were used. Nearest‐Neighbour Interchange was used as the ML heuristic method and the initial tree was made automatically. The topology of the tree was tested with 100 bootstrap replicates. Table S1 and Method S1 give more information on the genes, databases and methods used.

### Examination of potential COMT genes for the presence of conserved residues for COMT function

The initial alignment (before Gblocks removal of poorly aligned regions) visualised with ESPript 3.0 (Robert and Gouet, 2014) is included as Figure S2. The sequences were examined for the presence of the conserved residues for COMT function characterised by Zubieta *et al*. (2002) in alfalfa (*Medicago sativa*) *MsCOMT*.

### Plant materials, growth conditions and designation of internodes

Barley (*H. vulgare* ssp. *vulgare* cv. Golden Promise) was grown in a greenhouse with supplementary lighting from high pressure sodium vapour lamps. Plants for root sampling were grown in 50 : 50 sand and perlite. The internode nearest the crown greater than 1 cm long was designated the first internode, as in Tottman (1987).

### Crude protein extraction and quantification

An appropriate tissue and developmental stage to assay was determined by investigating *O*‐methylation of caffeic acid in internodes at different developmental stages (Figure S3a,b). Crude protein was extracted from 1 cm internode base by crushing in a 1.5 mL eppendorf in extraction buffer (100 mm Tris‐HCl pH 7.5, 20 mm β‐ME, 2% w/v PVPP, 2% w/v PEG, 1× Complete (Roche, UK)). Extracts were clarified by centrifugation and protein concentrations determined (Bradford, 1976) using the Bio‐Rad reagent (Bio‐Rad) and BSA standard.

### Caffeic acid O‐methyltransferase enzyme assay

The protocol of Fukuda and Komamine (1982) was used with modifications. Crude protein was incubated in 300 μL reactions containing 1.2 KBq S‐adenosyl‐^14^C‐methyl‐l‐methionine (SAM) (Perkin Elmer, MA), 100 mm sodium ascorbate, 10 mm MgCl_2_, 1 mm caffeic acid (Sigma, UK) and 0.1 M potassium phosphate pH 7.5, and incubated at 30 °C for either 30 min or 3 h. The radioactive product was extracted with ethyl acetate and measured using a TriCarb 3100 TR scintillation analyser (Packard, CT).

### COMT expression analysis in RNAi lines

For real‐time PCR analysis, total RNA was extracted from internodes with the Plant RNA Reagent (Invitrogen, UK) and cleaned‐up by DNAase treatment on an RNeasy column (Qiagen, UK) before further DNase treatment of the eluent with Turbo DNase (Ambion, CA). RNA was checked via Nanodrop and the Bioanalyzer 2100 (Agilent, UK). cDNA was synthesised from 600 ng RNA with random primers using iscript reverse transcriptase (Bio‐Rad, UK). Barley homologues of wheat genes *TaSnRK1* (Gene Index TC253257) and *TaRPII36* (Gene Index TC235230) (Kam *et al*., 2007) named here as *HvSnRK1* and *HvRPII36* were used as reference genes. Tables S2 and S3 give primer sequences and reaction set up. PCR products were validated by sequencing. Three technical replicates were performed for each gene and sample. Relative expression was calculated with the Pfaffl efficiency equation (Pfaffl, 2001) using the primer efficiency determined by LinRegPCR in the equation.

### Generation of RNAi construct and barley transformation

Primers containing Gateway AttB sites (Table S2) amplified a 634 bp fragment of *HvCOMT1* from Golden Promise cDNA which was recombined into pIPKb007 (Himmelbach *et al*., 2007) according to Invitrogen's instructions. Barley cv. Golden Promise was transformed via *Agrobacterium tumefaciens* AGL1 using the John Innes Centre (JIC) barley transformation protocol (Harwood *et al*., 2008) at JHI's Fungen facility. Southern analysis identified nine lines containing a single T‐DNA locus (Figure S4; Method S4). Zygosity was determined with the hygromycin root assay (Jacobsen *et al*., 2006).

### PAGE and western blotting

Denatured crude protein was separated by SDS‐PAGE on 4%–12% NuPage^®^ Bis‐Tris precast gels (Invitrogen) (roots) or 10% homemade acrylamide gels (internodes). Proteins were electroblotted onto Amersham Hybond ECL nitrocellulose membranes (GE Healthcare, UK). Membranes were blocked with 5% w/v milk powder in tris buffered saline pH 7.5, 0.1% v/v Tween‐20, washed, incubated with primary antibody (1 : 10 000), washed, incubated with HRP‐conjugated anti‐rabbit IgG (1 : 10 000) (NEB, UK), detected using LumiGLO^®^ and Peroxide Reagents (NEB) and visualized with Amersham Hyperfilm ECL (GE Healthcare). The generation of a recombinant HvCOMT1 to raise antibodies is described in Method S3.

### Klason lignin and thioacidolysis

T1 generation straw (leaves removed) were ground to pass a 0.5 mm screen. Extract‐free samples were prepared by exhaustive extraction with water, then ethanol. Klason lignin was measured according to Dence (1992). Lignin structure was evaluated by thioacidolysis followed by gas chromatography‐mass spectrometry (GC‐MS) of lignin‐derived monomers analysed as their trimethylsilyl derivatives (Lapierre *et al*., 1999; Rolando *et al*., 1992). The thioacidolysis compounds derived from *p*‐coumaric or ferulic acid (i.e. free acid and its EtSH addition product) were also quantified to evaluate the amount of cell wall‐linked *p*‐coumaric and ferulic units.

### Cell wall characterization by two‐dimensional solution‐state NMR

Cell walls were characterised without fractionation using two‐dimensional (2D) solution‐state NMR (Kim and Ralph, 2010; Mansfield *et al*., 2012). Straw (2‐mm pieces) was pre‐ground using a Mixer Mill MM400 (Retsch; 30/s vibrational frequency for 90–120 s). Samples were extracted three times with water, three times with 80% ethanol and once with acetone, then allowed to dry. The pre‐ground extracted samples were ball‐milled using a Fritsch Planetary micro mill Pulverisette 7 vibrating at 600 rpm with zirconium dioxide (ZrO_2_) vessels containing ZrO_2_ ball bearings (10 mm × 10) with 5‐min milling and a 5‐min cooling per milling cycle (cycle number depended on the amount of sample). The ball‐milled samples were subjected to digestion (72 h × 2) to obtain ‘enzyme lignin’ (EL) by Cellulysin^®^ Cellulase, *Trichoderma viridae* (Calbiochem), at 35 °C in acetate buffer (pH 5.0). The EL were dissolved into DMSO‐d_6_/pyridine‐d_5_ (4 : 1) and subjected to NMR on a Bruker Biospin AVANCE‐III 700 MHz spectrometer equipped with a 5‐mm QCI ^1^H/^31^P/^13^C/^15^N cryoprobe with inverse geometry (proton coil closest to the sample). 2D‐^1^H‐^13^C HSQC spectra were acquired using Bruker's pulse program (hsqcetgpsip2.2). Bruker's Topspin 3.2 (Mac) software was used to process spectra. The central DMSO peak was used as internal references (δ_C_: 39.51, δ_H_: 2.49 ppm).

### Transcript and metabolite profiling

Five plants per line were grown for 61 days in a randomised block design. The bottom three internodes were collected, frozen and ground in liquid nitrogen, and each sample divided into two aliquots, one for transcriptome analysis and one for metabolite analysis. See Method S5 and S6 for full details.

### Saccharification analyses

The same extracted sample used for lignin analysis (30 mg) was pretreated with 450 μL 1% w/v sulphuric acid in an autoclave (Astell, UK) at 121 °C for 1 h in 2 mL tubes or saccharified without pretreatment. Solids were washed three times with 1.5 mL 25 mm sodium acetate pH 4.5. Saccharifying enzyme mixture (Celluclast and Novozyme 188 (Sigma)) was prepared as described in Gomez *et al*. (2010). The FPU (filter paper unit) activity (65 FPU/mL) of the purified mixture was measured (Adney and Baker, 1996) along with β‐glucosidase activity (95.7 CBU/mL) (Ghose, 1987). Saccharification was performed with an enzyme loading of 0.6 FPU per 30 mg of sample in 25 mm sodium acetate pH 4.5 with 0.02% w/v NaN_3_ in a total volume of 1.5 mL for 72 h at 50 °C with shaking. Triplicate reactions were performed per plant. Glucose released was quantified using the GOPOD assay kit (K‐GLUC) (Megazyme, Ireland) scaled for a 96‐well plate and expressed as a proportion of the 30 mg extracted sample.

### Statistical analysis

For most analyses, a Student's *t*‐test was used in Excel (Microsoft) with the option for unequal variances selected where sample sizes differed. For the metabolomics/transcriptomic experiment, model‐adjusted means were used. Only metabolites/probes whose combined mean was at least threefold and significantly (*P* < 0.01) different from combined controls in both COMT RNAi lines was considered to be meaningfully different.

## Conflict of interest

The authors declare no conflict of interest.

## Supporting information


**Figure S1** The Gblocks selected parts of the original alignment used to construct the phylogenetic tree for Figure 1.

**Figure S2** Alignment of the genes from the phylogenetic analysis demonstrating the absence or presence of conserved residues for COMT function.

**Figure S3** Investigation of *O*‐methylation of caffeic acid in barley internodes at different developmental stages; one of the biological replicate plants from the succession sampled at each time point is shown with the number of weeks after sowing indicated.
**Figure S4** Southern blot analysis for T‐DNA locus number of the COMT lines which had reduced enzyme activity.
**Figure S5** The expression of *HvCOMT1* and *HvCOMT2* genes in internodes at different developmental stages.
**Figure S6** Gene expression levels for all of the barley genes from the phylogenetic analysis for which data were available in a 16‐tissue RNAseq dataset described by Mascher *et al*. (2017).
**Figure S7** Biomass measurements of COMT RNAi lines.
**Figure S8** Correlation between the amounts of p‐coumaric acid (CA) released by thioacidolysis and mild alkaline hydrolysis.
**Figure S9** MS‐based structural elucidation of the differentially accumulating m/z traces in COMT RNAi lines as compared to empty vector and wild‐type controls.

**Table S1** (Excel file) Further information on the genes from the phylogenetic analysis in Figure 1
**Table S2** Primer sequences used in experiments in this study
**Table S3** Summary of reaction set‐up and cycling conditions for real‐time PCR
**Table S4** The number of the conserved residues present for the binding/positioning of COMT substrates ferulic acid and 5‐hydroxyconiferaldehyde, as identified by (Zubieta *et al*., 2002) in MsCOMT
**Table S5** Demonstration of the shared synteny between the barley and wheat chromosome arms that the barley COMT genes and homologous wheat gene(s) map to
**Table S6** Summary of the source tissue of ESTs for HvCOMT1, HvCOMT2 and HvCOMT3 from HarvEST #35
**Table S7** Summary of lignin data from the COMT RNAi lines

**Table S8** m/z traces with a different intensity in the internodes of COMT RNAi lines as compared to empty vector and wild‐type controls
**Table S9** Microarray transcriptome analysis of internodes from two COMT RNAi lines (COMTRNAi_4 and COMTRNAi_14) and controls (empty vector and wild‐type)
**Method S1** Sequence retrieval, data sources and multiple alignment
**Method S2** Determination of the genomic location and evidence for expression of barley genes
**Method S3** Generation and purification of a recombinant HvCOMT1 protein for antibody production
**Method S4** Southern Blot
**Method S5** RNA extraction and microarray processing for transcriptome analysis
**Method S6** Phenolic metabolome analysis
