# Supplementary material for: RNAi‐suppression of barley caffeic acid O‐methyltransferase modifies lignin despite redundancy in the gene family
Source: Plant Biotechnol J. 2018 Oct 2;17(3):594–607. doi: 10.1111/pbi.13001 (PMC6381794; doi:10.1111/pbi.13001)
Supplement: Supplementary file 2 — Figure S2 Alignment of the genes from the phylogenetic analysis demonstrating the absence or presence of conserved residues for COMT function. [file PBI-17-594-s003.pdf]

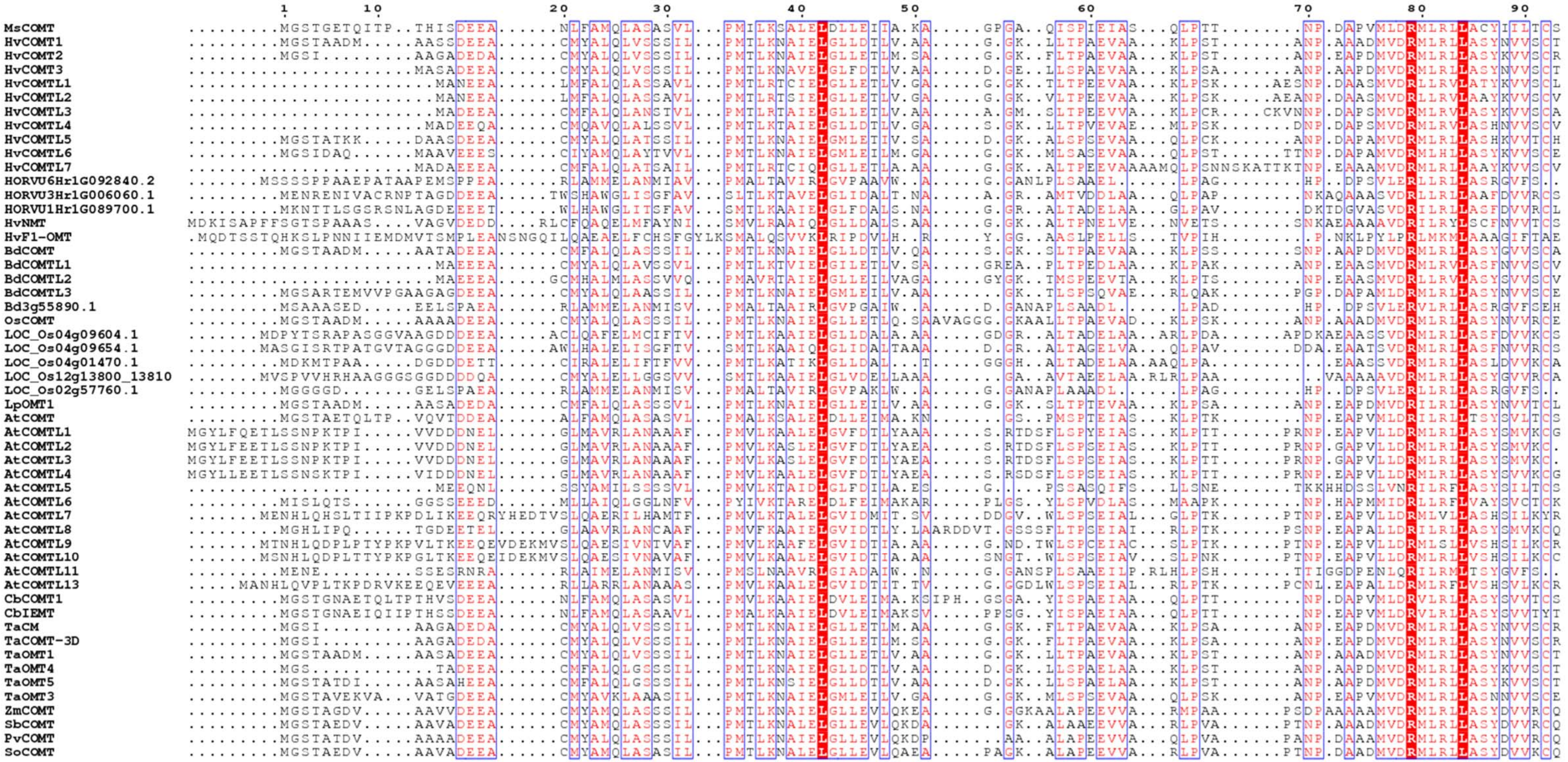
1
10
20
30
40
50
60
70
80
90
MsCOMT
HvCOMT1
HvCOMT2
HvCOMT3
HvCOMTL1
HvCOMTL2
HvCOMTL3
HvCOMTL4
HvCOMTL5
HvCOMTL6
HvCOMTL7
HORVU6Hr1G092840.2
HORVU3Hr1G006060.1
HORVU1Hr1G089700.1
HvNMT
HvF1-OMT
BdCOMT
BdCOMTL1
BdCOMTL2
BdCOMTL3
Bd3g55890.1
OsCOMT
LOC_Os04g09604.1
LOC_Os04g09654.1
LOC_Os04g01470.1
LOC_Os12g13800_13810
LOC_Os02g57760.1
LpOMT1
AtCOMT
AtCOMTL1
AtCOMTL2
AtCOMTL3
AtCOMTL4
AtCOMTL5
AtCOMTL6
AtCOMTL7
AtCOMTL8
AtCOMTL9
AtCOMTL10
AtCOMTL11
AtCOMTL13
CbCOMT1
CbIEMT
TaCM
TaCOMT-3D
TaOMT1
TaOMT4
TaOMT5
TaOMT3
ZmCOMT
SbCOMT
PvCOMT
SoCOMT

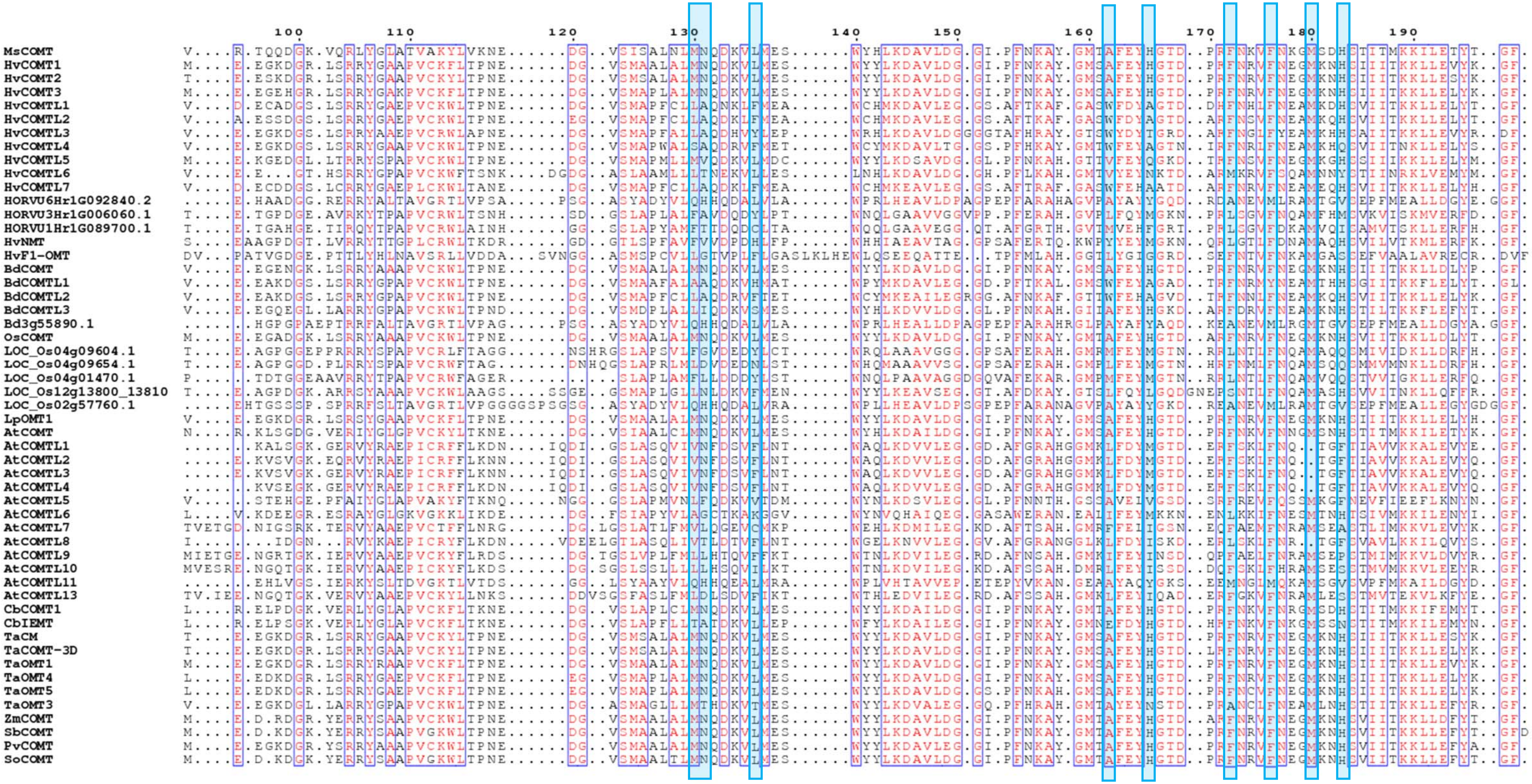

100
110
120
130
140
150
160
170
180
190
MsCOMT
HvCOMT1
HvCOMT2
HvCOMT3
HvCOMTL1
HvCOMTL2
HvCOMTL3
HvCOMTL4
HvCOMTL5
HvCOMTL6
HvCOMTL7
HORVU6Hr1G092840.2
HORVU3Hr1G006060.1
HORVU1Hr1G089700.1
HvNMT
HvF1-OMT
BdCOMT
BdCOMTL1
BdCOMTL2
BdCOMTL3
Bd3g55890.1
OsCOMT
LOC_Os04g09604.1
LOC_Os04g09654.1
LOC_Os04g01470.1
LOC_Os12g13800_13810
LOC_Os02g57760.1
LpOMT1
AtCOMT
AtCOMTL1
AtCOMTL2
AtCOMTL3
AtCOMTL4
AtCOMTL5
AtCOMTL6
AtCOMTL7
AtCOMTL8
AtCOMTL9
AtCOMTL10
AtCOMTL11
AtCOMTL13
CbCOMT1
CbIEMT
TaCM
TaCOMT-3D
TaOMT1
TaOMT4
TaOMT5
TaOMT3
ZmCOMT
SbCOMT
PvCOMT
SoCOMT

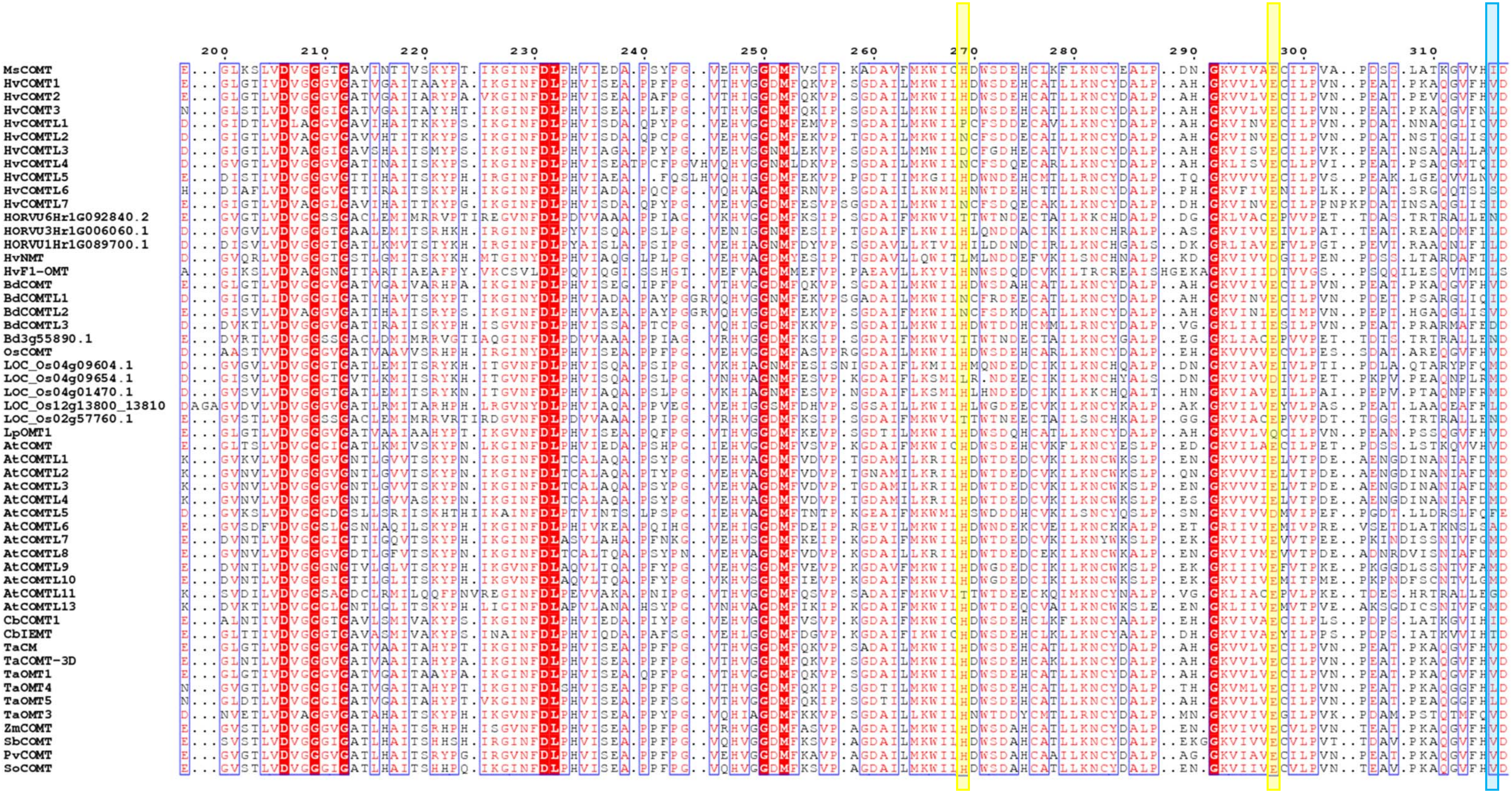

200
210
220
230
240
250
260
270
280
290
300
310
MsCOMT
HvCOMT1
HvCOMT2
HvCOMT3
HvCOMTL1
HvCOMTL2
HvCOMTL3
HvCOMTL4
HvCOMTL5
HvCOMTL6
HvCOMTL7
HORVU6Hr1G092840.2
HORVU3Hr1G006060.1
HORVU1Hr1G089700.1
HvNMT
HvF1-OMT
BdCOMT
BdCOMTL1
BdCOMTL2
BdCOMTL3
Bd3g55890.1
OsCOMT
LOC_Os04g09604.1
LOC_Os04g09654.1
LOC_Os04g01470.1
LOC_Os12g13800_13810
LOC_Os02g57760.1
LpOMT1
AtCOMT
AtCOMTL1
AtCOMTL2
AtCOMTL3
AtCOMTL4
AtCOMTL5
AtCOMTL6
AtCOMTL7
AtCOMTL8
AtCOMTL9
AtCOMTL10
AtCOMTL11
AtCOMTL13
CbCOMT1
CbIEMT
TaCM
TaCOMT-3D
TaOMT1
TaOMT4
TaOMT5
TaOMT3
ZmCOMT
SbCOMT
PvCOMT
SoCOMT

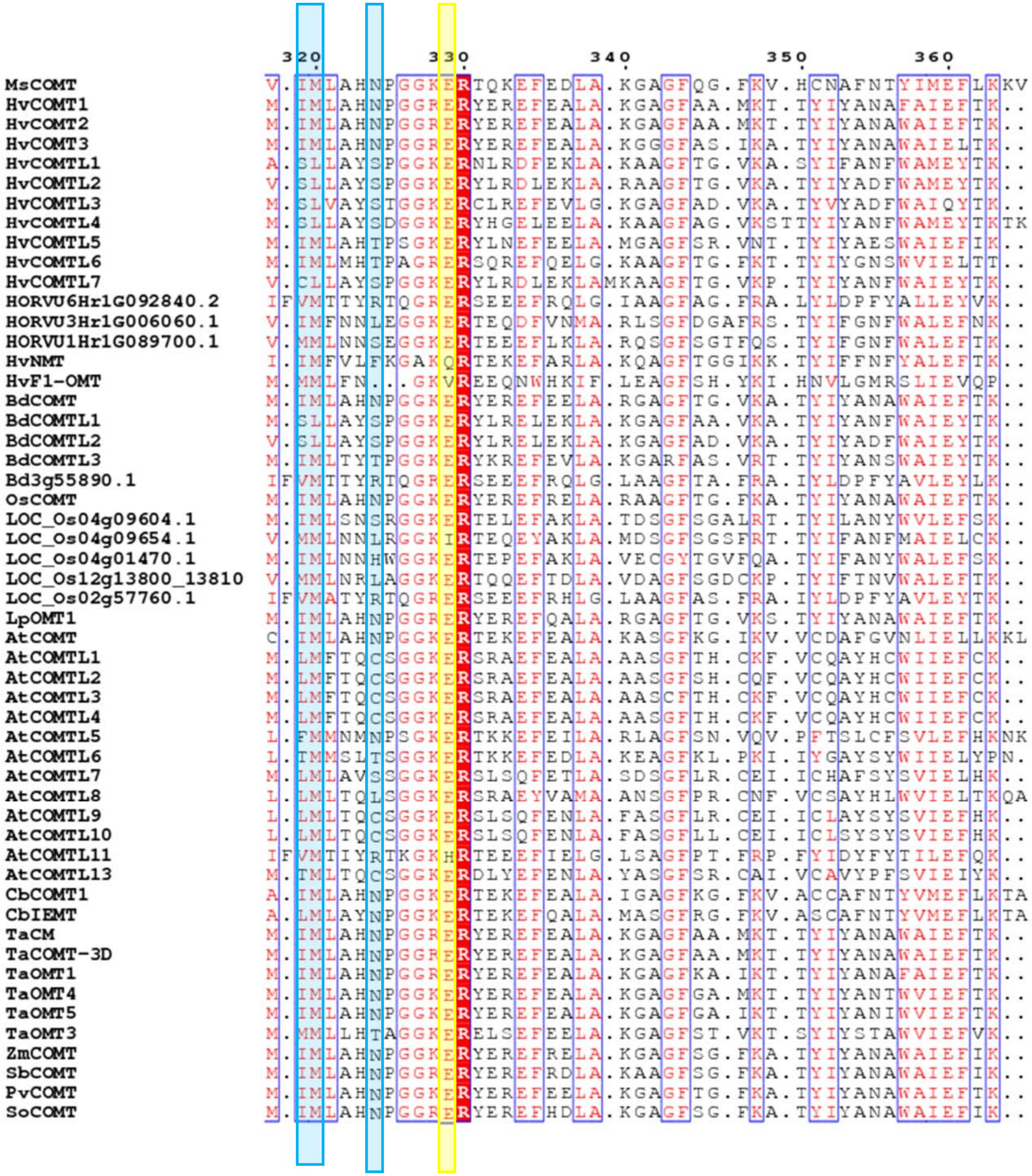

320 330 340 350 360
MsCOMT V.IMLAHNPGGKERTQKEFEDLA.KGAGFQG.FKV.HCNAFNTYIMEFLKKV
HvCOMT1 M.IMLAHNPGGRERYEREFEALA.KGAGFAA.MKT.TYIYANAFAIEFTK..
HvCOMT2 M.IMLAHNPGGRERYEREFEALA.KGAGFAA.MKT.TYIYANAWAIEFTK..
HvCOMT3 M.IMLAHNPGGRERYEREFEALA.KGGGFAS.IKA.TYIYANAWAIELTK..
HvCOMTL1 A.SLLAYSPGGKERNLRDFEKLA.KAAGFTG.VKA.SYIFANFWAMEYTK..
HvCOMTL2 V.SLLAYSPGGKERYLRDLEKLA.RAAGFTG.VKA.TYIYADFWAMEYTK..
HvCOMTL3 M.SLVAYSTGGKERCLREFEVLG.KGAGFAD.VKA.TYVYADFWAIQYTK..
HvCOMTL4 M.SLLAYSDGGKERYHGELEELA.KAAGFAG.VKSTTYIYANFWAMEYTKTK
HvCOMTL5 M.IMLAHTPSGKERYLNEFEELA.MGAGFSR.VNT.TYIYAESWAIEFIK..
HvCOMTL6 M.IMLMHTPAGRERSQREFQELG.KAAGFTG.FKT.TYIYGNSWVIELTT..
HvCOMTL7 V.CLLAYSPGGKERYLRDLEKLAMKAAGFTG.VKP.TYIYANFWAIEYTK..
HORVU6Hr1G092840.2 IFVMTTYRTQGRERSEEEFRQLG.IAAGFAG.FRA.LYLDPFYALLEYVK..
HORVU3Hr1G006060.1 V.IMFNNLEGGKERTEQDFVNMA.RLSGFDGAFRS.TYIFGNFWALEFNK..
HORVU1Hr1G089700.1 V.MMLNNSEGGKERTEEEFLKLA.RQSGFSGTFQS.TYIFGNFWALEFTK..
HvNMT I.IMFVLFKGAKQRTEKEFARLA.KQAGFTGGIKK.TYIFFNFYALEFTK..
HvF1-OMT M.MMLFN...GKVREEQNWHKIF.LEAGFSH.YKI.HNVLGMRSLIEVQP..
BdCOMT M.IMLAHNPGGKERYEREFEELA.RGAGFTG.VKA.TYIYANAWAIEFTK..
BdCOMTL1 M.SLLAYSPGGKERYLRELEKLA.KGAGFAA.VKA.TYIYANFWAIEYTK..
BdCOMTL2 V.SLLAYSPGGKERYLRELEKLA.KGAGFAD.VKA.TYIYADFWAIEYTK..
BdCOMTL3 M.IMLTYTPGGKERYKREFEVLA.KGARFAS.VRT.TYIYANSWAIEYTK..
Bd3g55890.1 IFVMTTYRTQGRERSEEEFRQLG.LAAGFTA.FRA.IYLDPFYAVLEYLK..
OsCOMT M.IMLAHNPGGKERYEREFRELA.RAAGFTG.FKA.TYIYANAWAIEFTK..
LOC_Os04g09604.1 M.IMLSNSRGGKERTELEFAKLA.TDSGFSGALRT.TYILANYWVLEFSK..
LOC_Os04g09654.1 V.MMLNNLRGGKIRTEQEYAKLA.MDSGFSGSFRT.TYIFANFMAIELCK..
LOC_Os04g01470.1 M.IMLNNHWGGKERTEPEFAKLA.VECGYTGVFQA.TYIFANYWALEFSK..
LOC_Os12g13800_13810 V.MMLNRLAGGKERTQQEFTDLA.VDAGFSGDCKP.TYIFTNVWALEFTK..
LOC_Os02g57760.1 IFVMATYRTQGRERSEEEFRHLG.LAAGFAS.FRA.IYLDPFYAVLEYTK..
LpOMT1 M.IMLAHNPGGRERYEREFQALA.RGAGFTG.VKS.TYIYANAWAIEFTK..
AtCOMT C.IMLAHNPGGKERTEKEFEALA.KASGFKG.IKV.VCDAFGVNLIELLKKL
AtCOMTL1 M.LMFTQCSGGKERSRAEFEALA.AASGFTH.CKF.VCQAYHCWIIEFCK..
AtCOMTL2 M.LMFTQCSGGKERSRAEFEALA.AASGFSH.CQF.VCQAYHCWIIEFCK..
AtCOMTL3 M.LMFTQCSGGKERSRAEFEALA.AASCFTH.CKF.VCQAYHCWIIEFCK..
AtCOMTL4 M.LMFTQCSGGKERSRAEFEALA.AASGFTH.CKF.VCQAYHCWIIEFCK..
AtCOMTL5 L.FMMNMNPSGKERTKKEFEILA.RLAGFSN.VQV.PFTSLCFSVLEFHKNK
AtCOMTL6 L.TMMSLTSGGKERTKKEFEDLA.KEAGFKL.PKI.IYGAYSYWIIELYPN.
AtCOMTL7 M.LMLAVSSGGKERSLSQFETLA.SDSGFLR.CEI.ICHAFSYSVIELHK..
AtCOMTL8 L.LMLTQLSGGKERSRAEYVAMA.ANSGFPR.CNF.VCSAYHLWVIELTKQA
AtCOMTL9 L.LMLTQCSGGKERSLSQFENLA.FASGFLR.CEI.ICLAYSYSVIEFHK..
AtCOMTL10 L.LMLTQCSGGKERSLSQFENLA.FASGFLL.CEI.ICLSYSYSVIEFHK..
AtCOMTL11 IFVMTIYRTKGKHRTEEEFIELG.LSAGFPT.FRP.FYIDYFYTILEFQK..
AtCOMTL13 M.TMLTQCSGGKERDLYEFENLA.YASGFSR.CAI.VCAVYPFSVIEIYK..
CbCOMT1 A.IMLAHNPGGKERTEKEFEALA.IGAGFKG.FKV.ACCAFNTYVMEFLKTA
CbIEMT A.IMLAYNPGGKERTEKEFQALA.MASGFRG.FKV.ASCAFNTYVMEFLKTA
TaCM M.IMLAHNPGGRERYEREFEALA.KGAGFAA.MKT.TYIYANAWAIEFTK..
TaCOMT-3D M.IMLAHNPGGRERYEREFEALA.KGAGFAA.MKT.TYIYANAWAIEFTK..
TaOMT1 M.IMLAHNPGGRERYEREFEALA.KGAGFKA.IKT.TYIYANAFAIEFTK..
TaOMT4 M.IMLAHNPGGKERYEREFEALA.KGAGFGA.MKT.TYIYANTWVIEFTK..
TaOMT5 M.IMLAHNPGGKERYEREFEALA.KGAGFGA.IKT.TYIYANIWVIEFTK..
TaOMT3 M.MMLLHTAGGKERELSEFEELA.KGAGFST.VKT.SYIYSTAWVIEFVK..
ZmCOMT M.IMLAHNPGGKERYEREFRELA.KGAGFSG.FKA.TYIYANAWAIEFIK..
SbCOMT M.IMLAHNPGGRERYEREFRDLA.KAAGFSG.FKA.TYIYANAWAIEFIK..
PvCOMT M.IMLAHNPGGKERYEREFEELA.KGAGFTG.FKA.TYIYANAWAIEFTK..
SoCOMT M.IMLAHNPGGRERYEREFHDLA.KGAGFSG.FKA.TYIYANAWAIEFIK..
