# Supplementary material for: RNAi‐suppression of barley caffeic acid O‐methyltransferase modifies lignin despite redundancy in the gene family
Source: Plant Biotechnol J. 2018 Oct 2;17(3):594–607. doi: 10.1111/pbi.13001 (PMC6381794; doi:10.1111/pbi.13001)
Supplement: Supplementary file 3 — Figure S3 Investigation of O‐methylation of caffeic acid in barley internodes at different developmental stages; one of the biological replicate plants from the succession sampled at each time point is shown with the number of weeks after sowing indicated. Figure S4 Southern blot analysis for T‐DNA locus number of the COMT lines which had reduced enzyme activity. Figure S5 The expression of HvCOMT1 and HvCOMT2 genes in internodes at different developmental stages. Figure S6 Gene expression levels for all of the barley genes from the phylogenetic analysis for which data were available in a 16‐tissue RNAseq dataset described by Mascher et al. (2017). Figure S7 Biomass measurements of COMT RNAi lines. Figure S8 Correlation between the amounts of p‐coumaric acid (CA) released by thioacidolysis and mild alkaline hydrolysis. Figure S9 MS‐based structural elucidation of the differentially accumulating m/z traces in COMT RNAi lines as compared to empty vector and wild‐type controls. [file PBI-17-594-s001.docx]

## Supporting Information

Fig. S 1. The Gblocks selected parts of the original alignment used to construct the phylogenetic tree for Fig. 1; poorly aligned regions were removed with Gblocks as described in the Experimental Procedures in the main article. (SEPARATE FILE .txt)

Fig. S 2. Alignment of the genes from the phylogenetic analysis demonstrating the absence or presence of conserved residues for COMT function. Residues highlighted by blue rectangles are substrate binding/positioning residues and yellow rectangles are catalytic residues for the COMT substrates ferulic acid and 5-hydroxyconiferaldehyde described by Zubieta et al. (2002) in *MsCOMT*. See Table S1 for further information on the genes in the alignment. (SEPARATE FILE .pdf)

Fig. S 3. Investigation of *O*-methylation of caffeic acid in barley internodes at different developmental stages. (a) Images of one of the biological replicate plants sampled at each time point from the succession is shown. The number of weeks after sowing and nodes present in the stem is indicated. This succession was grown in the winter season with the seeds for the oldest plants (12 weeks) in the succession sown on the 9^th^ of December. (b) Enzyme assay on crude extracts from internodes at different developmental stages. The graph shows the enzyme activity relative to the activity in the 2^nd^ internode eight weeks after sowing (as indicated by the black arrow). The error bars represent standard errors.


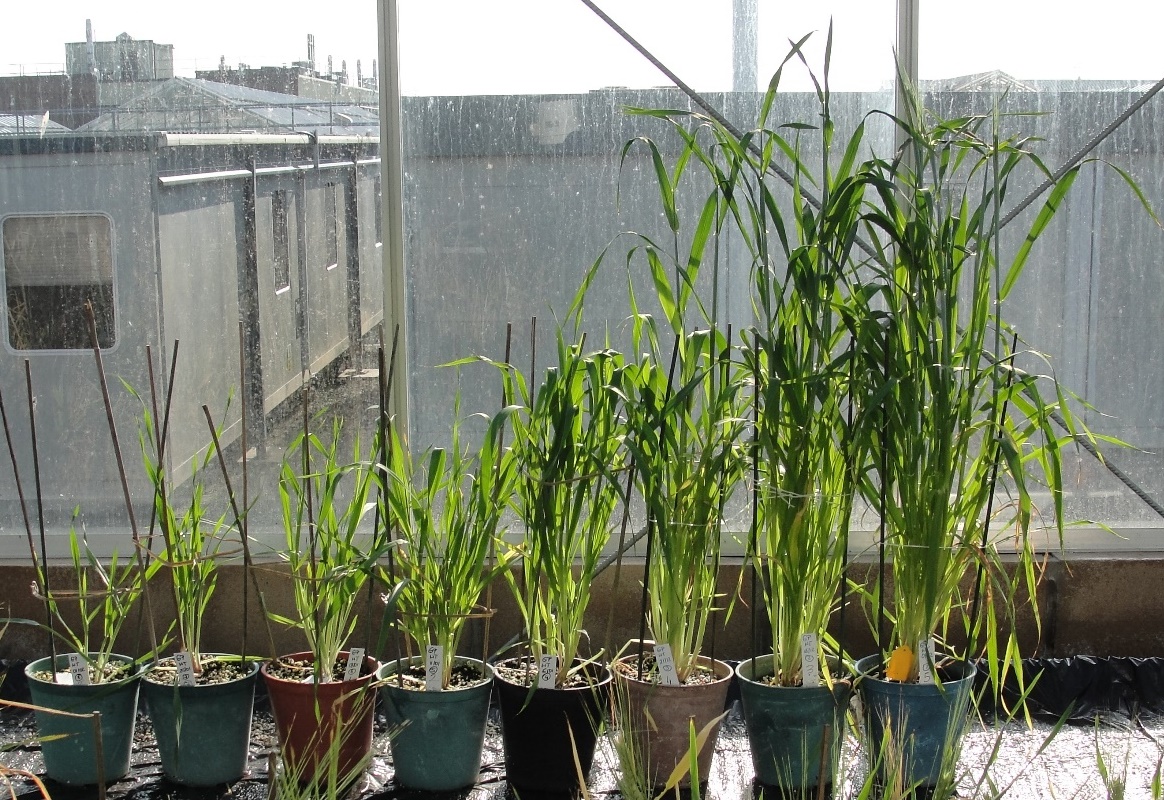


4 weeks 5 weeks 6 weeks 7 weeks 8 weeks 10 weeks 11 weeks 12 weeks

1 node 2 nodes 4 nodes 4 nodes 5-6 nodes 7 nodes 7 nodes 7 nodes

**(a)**

**(b)**

Fig. S 4. Southern blot analysis for T-DNA locus number of the COMT lines which had reduced enzyme activity.


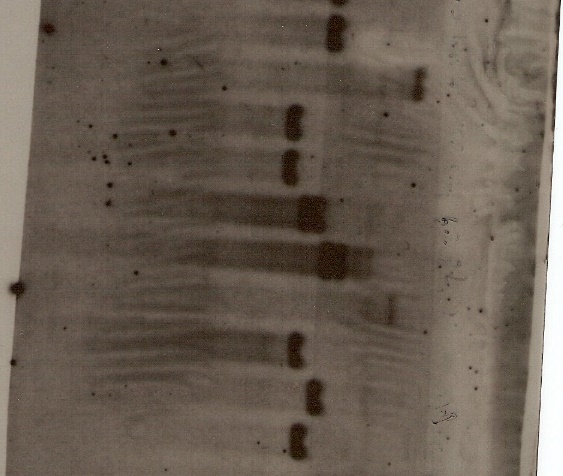

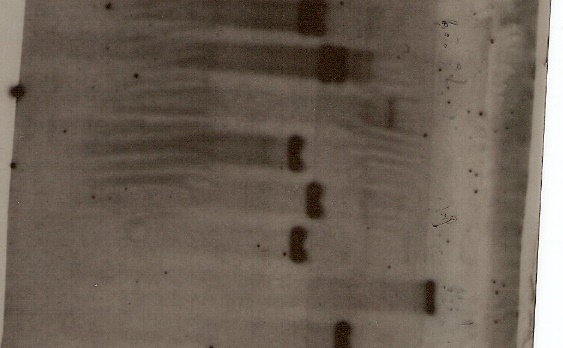

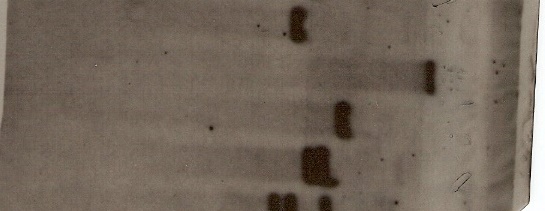


COMTRNAi_ lines

1 4 5 6 8 26 28 9 14 19 21

HindIII


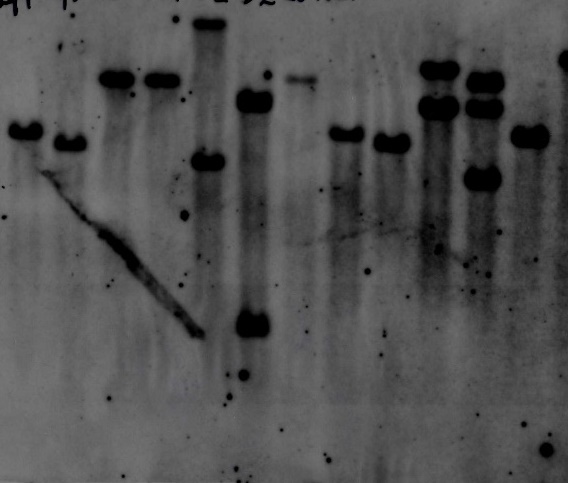

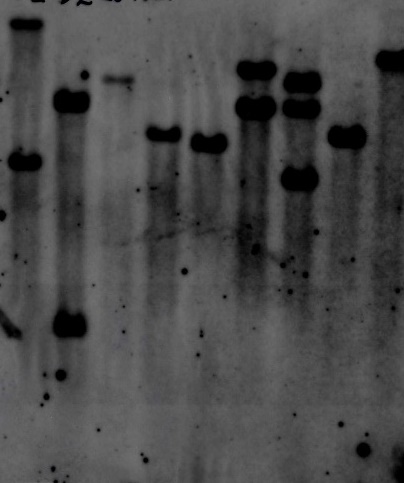

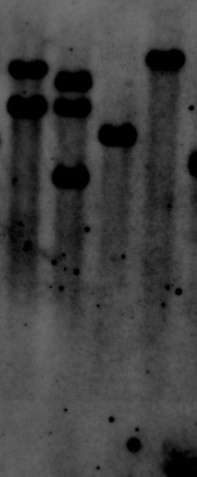


EcoRV

COMTRNAi_ lines

1 4 5 1 4 5 6 8 26 28 21 24 19 9 14


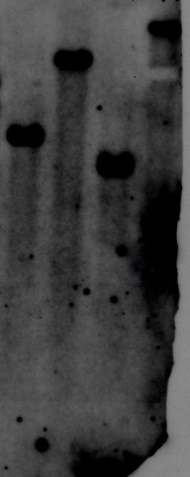


Fig. S 5. The expression of HvCOMT1 and HvCOMT2 genes in internodes at different developmental stages. The expression for each gene is relative to the expression of that gene in the internode beneath the peduncle when the flag leaf was emerging. Three plants were sampled at each developmental stage. The error bars represent standard errors.


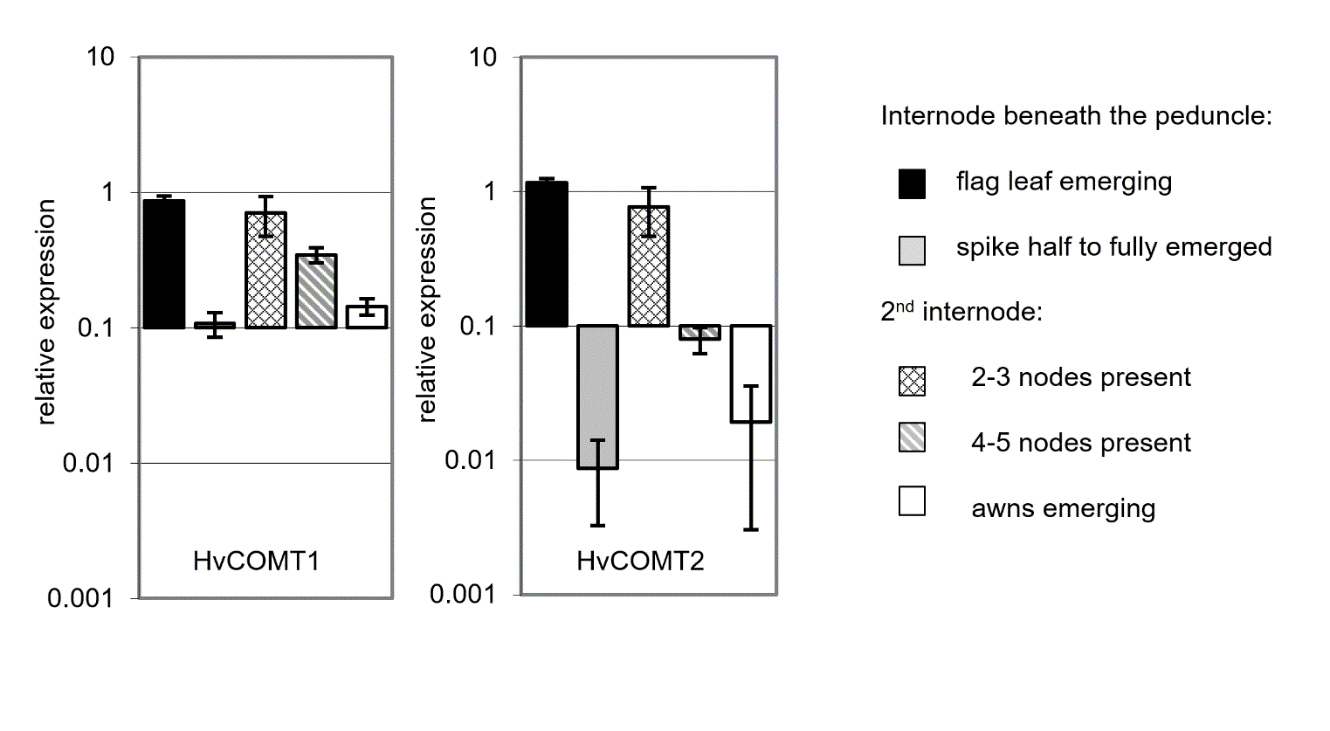


Fig. S 6. Gene expression levels for all of the barley genes from the phylogenetic analysis for which data was available in a 16-tissue RNAseq dataset described by Mascher et al. (2017). The expression values (FPKM) are the average of three biological replicates. The dataset was queried at the barleyGenes resource at https://ics.hutton.ac.uk/barleyGenes/ using the JLOC1 IDs listed in Table S 1. Abbreviations: EMB: 4-day embryos dissected from germinating grains, ROO1: Roots from the seedlings, LEA: Shoots from the seedlings, INF1: Young developing inflorescences (5mm), INF2: Developing inflorescences (1-1.5 cm), NOD: Developing tillers at six leaf stage, 3rd internode, CAR5: Developing grain, bracts removed (5 DPA), CAR15: Developing grain, bracts removed (15 DPA), ETI: Etiolated seedling (10 days), LEM: Lemma (6 weeks pa), LOD: Lodicule (6 weeks pa), PAL: Palea (6 weeks pa), EPI: Peeled epidermis (4 weeks), RAC: Rachis (5 weeks pa), ROO2: Root (4 weeks) and SEN: Senescing leaf (2 months).


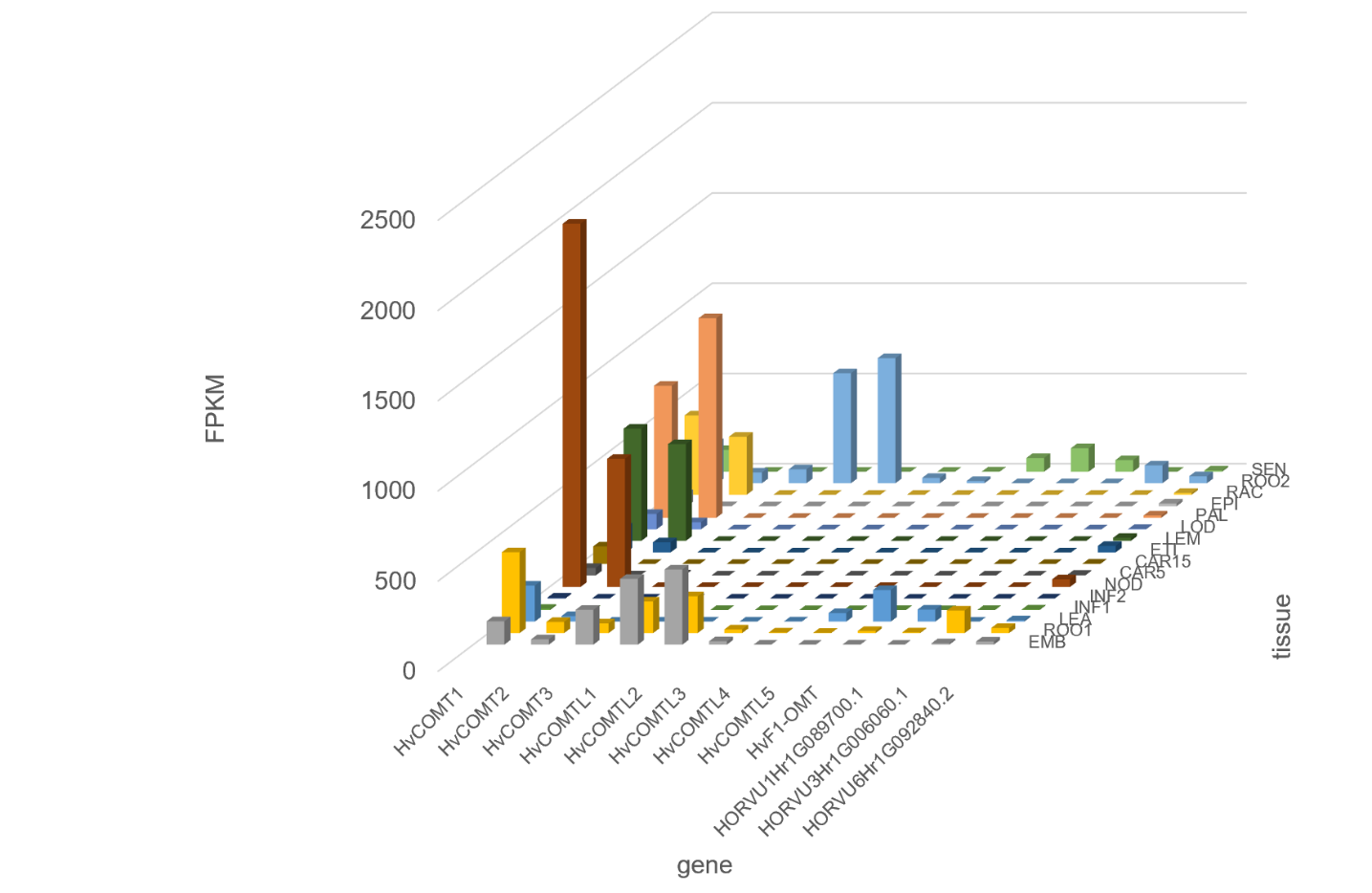


Fig. S 7. Biomass measurements of COMT RNAi lines from the (a) straw weight and (b) grain weight per plant. There was no significant difference between homozygotes and azygotes for any of the COMT RNAi lines for either measurement (Student’s *t*-test, *P*<0.05, n = 8). The error bars represent standard errors. The straw (all above ground parts of the plant except for the spike) was dried in an oven at 50°C before weighing.


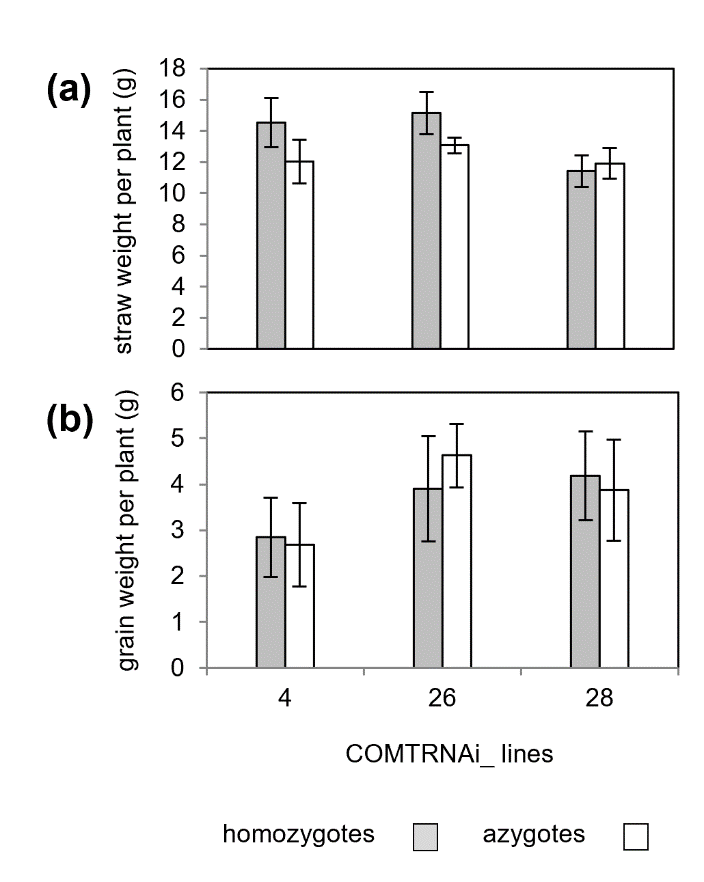


Fig. S 8. Correlation between the amounts of coumaric acid (CA) released by thioacidolysis and mild alkaline hydrolysis. The samples are extractive-free stem samples from barley, brachypodium, maize and rice prepared by exhaustive-extraction of ground stems by water, then ethanol before freeze-drying. This mild alkaline hydrolysis method is comprehensively described in Ho-Yue-Kuang et al., 2016. CW: cell wall.


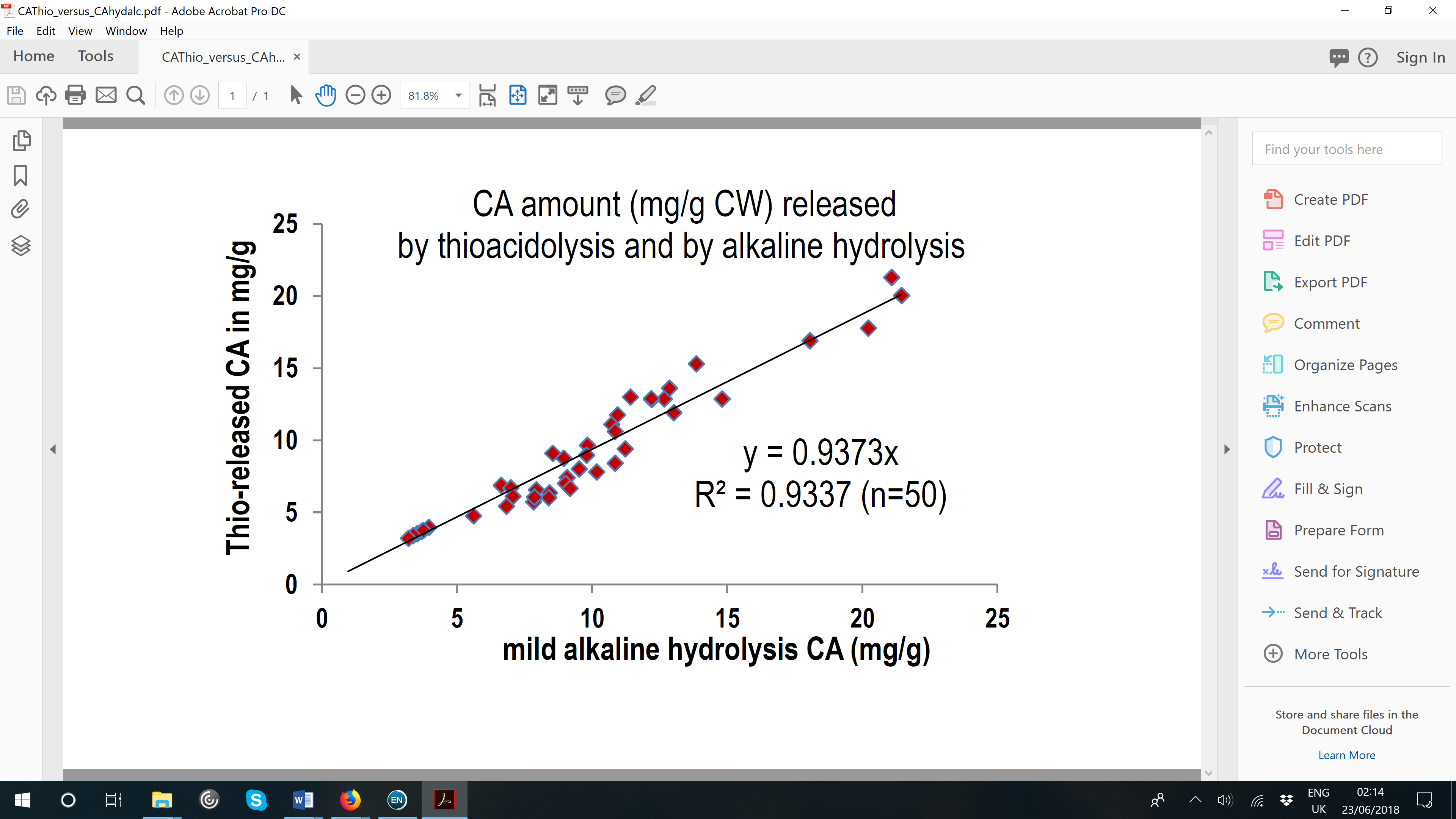


Fig. S 9. MS-based structural elucidation of the differentially accumulating m/z traces in COMT RNAi lines as compared to empty vector and wild type controls. Compound 1, G(8-O-4)5-OH-G, was characterized based on retention time of the molecular ion with m/z 373.129 and an in-source fragment with m/z 179.069 that match the authentic chemical standard. The spectrum shown is from the authentic chemical standard. Compound 2, S(8-O-4)5-OH-G, was characterized based on retention time of the molecular ion with m/z 403.140 and an in-source fragment with m/z 209.080 that match the authentic chemical standard. In addition, the MS/MS spectrum of m/z 209.080 matched with the MS/MS spectrum of sinapyl alcohol. The spectrum shown is from the authentic chemical standard. Compound 3 and 4 were characterized as S(8-O-4)G(8-O-4)5-OH-G isomers, based on their MS/MS fragmentation. The fragmentation of the S(8-O-4) moiety is as described by Morreel et al. (2010a) and Morreel et al. (2010b). The similarities with compound 2 are indicative for the S(8-O-4)5-OH-G moiety. The characterization of compound 5 is also based on its MS/MS fragmentation; an initial loss of 162.053 Da (resulting in m/z 195.066) hints the presence of hexose that is lost as dehydrated hexose. The observed fragments with m/z 195.066, 180.041, 177.067, 162.024 and 151.041 all match with the authentic chemical standard of 5-hydroxyconiferyl alcohol. Compound 6 is characterized based on the similarity with compound 5: the co-elution of the molecular ion with m/z 357.123 and the in-source fragment with m/z 195.066. No MS/MS spectrum could be obtained for this compound. The structural elucidation of compound 7 is based on its MS/MS fragmentation; initial neutral losses of 42.011 Da (resulting in m/z 357.166) and 204.063 Da (resulting in m/z 195.066) hints the presence of an acetyl and an acetylhexose, respectively. The observed fragments with m/z 195.064, 180.041, 177.052, 162.031 and 151.038 all match with the authentic chemical standard of 5-hydroxyconiferyl alcohol. Compound 8, caffeyl alcohol + hexose, is characterized based on its MS/MS fragmentation. An initial loss of 162.053 Da (resulting in m/z 165.053) hints the presence of hexose that is lost as dehydrated hexose. The observed fragments with m/z 165.053, 147.044, 129.031 and 101.027 match with the fragments of caffeyl alcohol. The structural elucidation of compound 9, is similar to the one of compound 8, except that the initial neutral loss is 204.063 (resulting in m/z 165.054), which is indicative for the presence of acetylhexose. Compound 10 and 11 were elucidated based on MS/MS as described in Tsuji et al. (2015) and Morreel et al. (2010a) and Morreel et al. (2010b).

See Legend for Fig. S 9 on previous page.


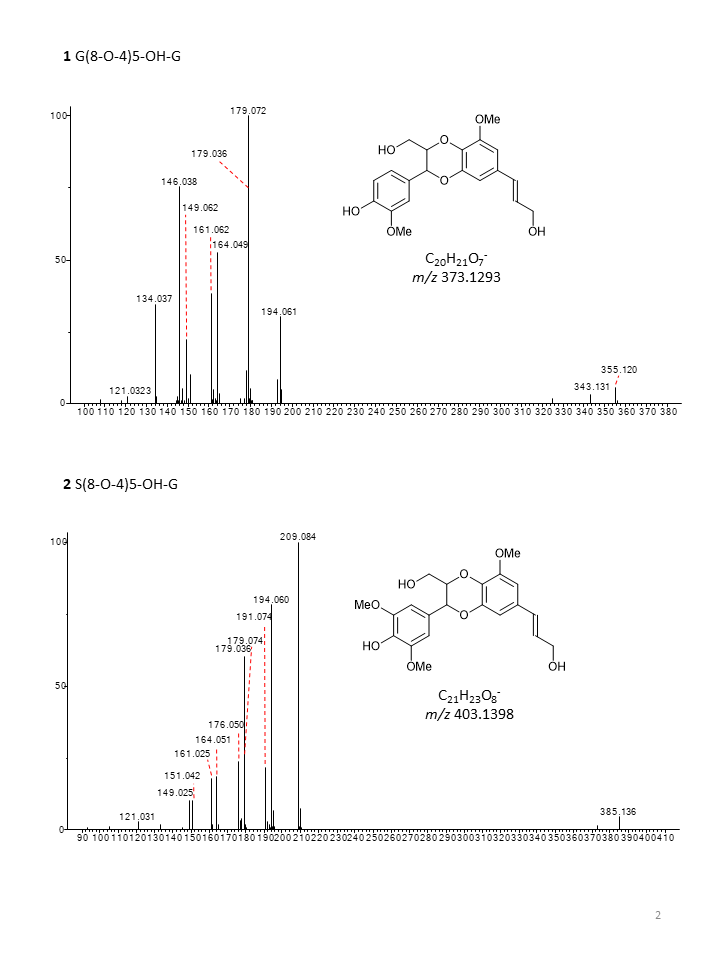

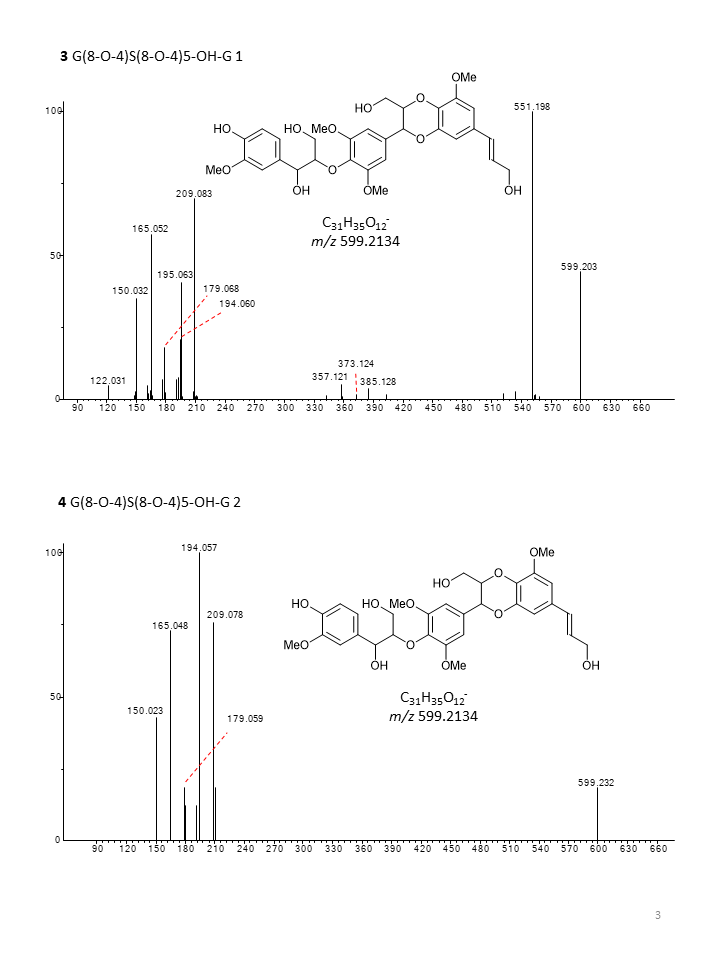


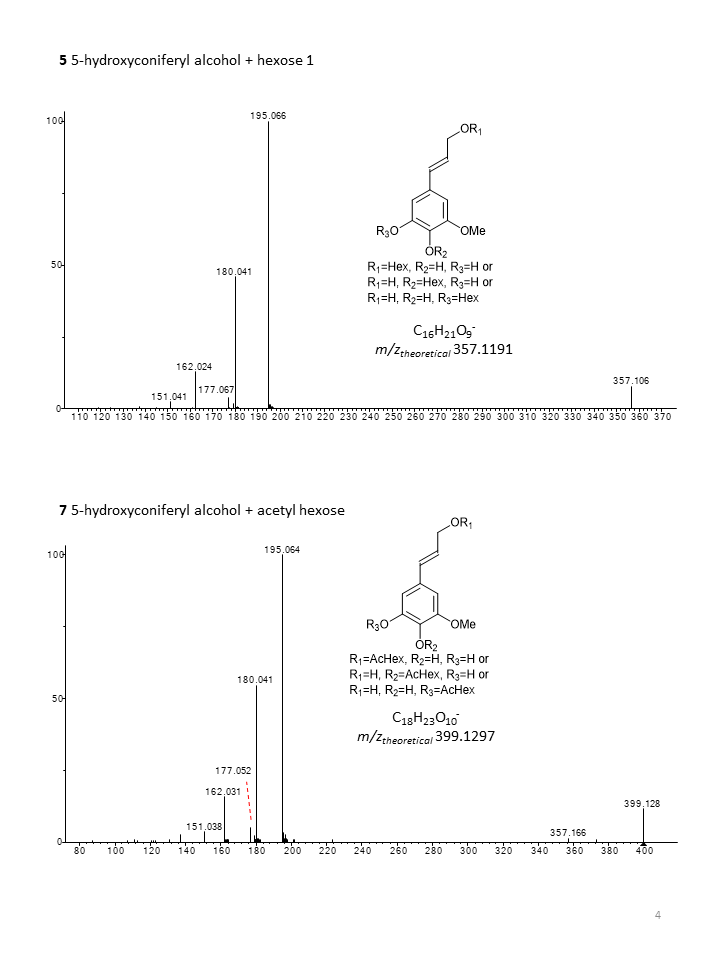

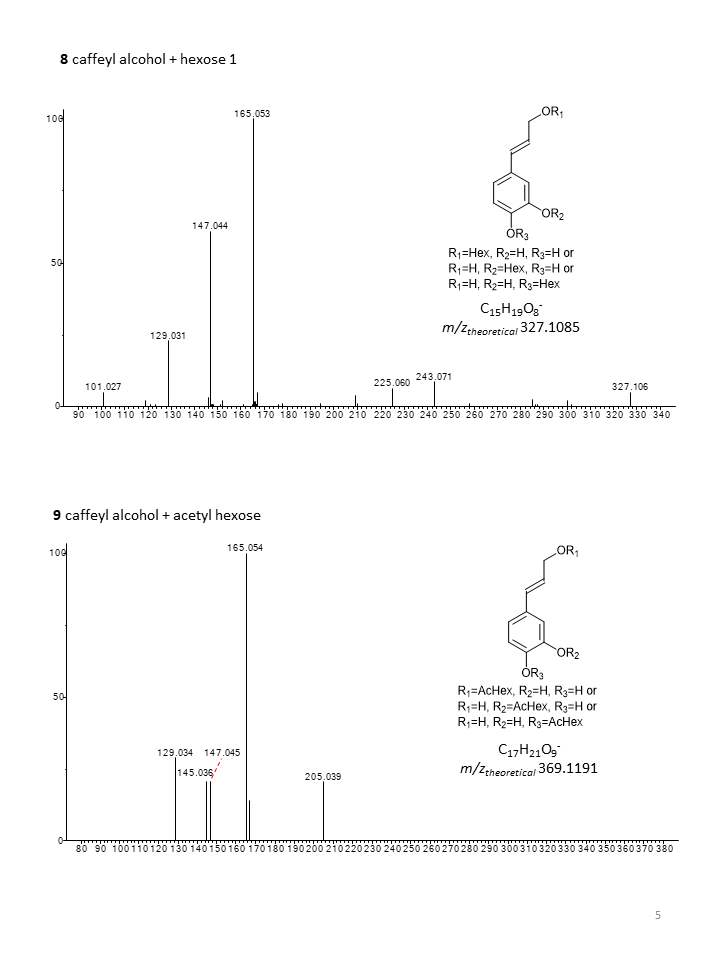


See Legend for Fig. S 9 on previous page.


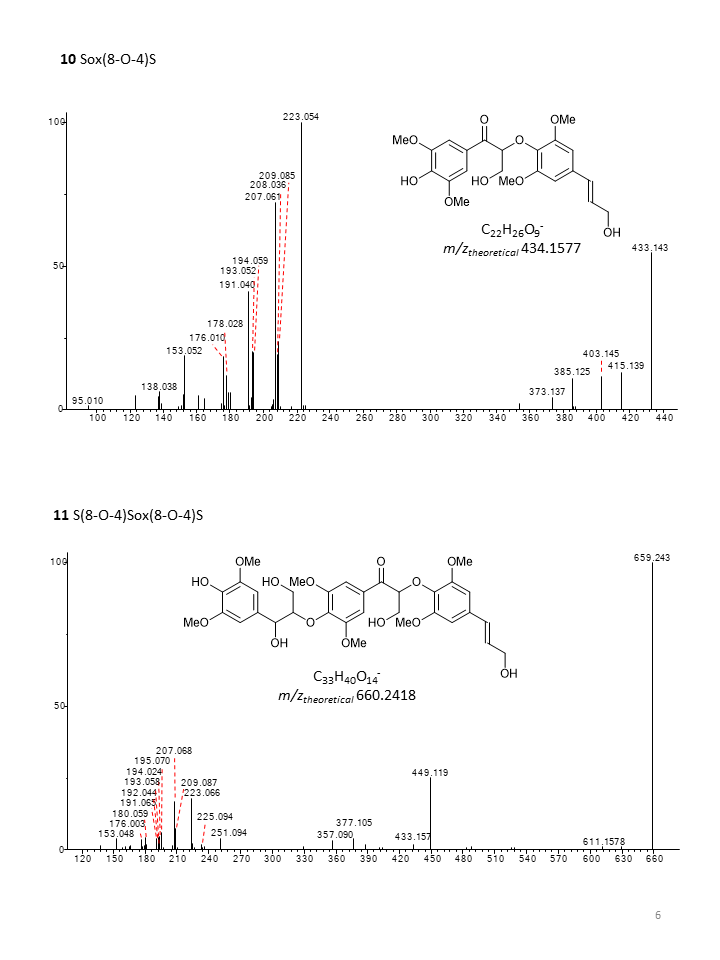


Table S 1. Further information on the genes from the phylogenetic analysis in Figure 1 and the alignments in Fig. S1 and Fig. S2. (SEPARATE FILE .xls)

Table S 2. Primer sequences used in experiments in this study. Sequences highlighted in bold are AttB and linker sequences used for the Gateway cloning method.

| experiment | gene | primer sequence |
| --- | --- | --- |
| real-time PCR | HvCOMT1 | Fw5′TGAGACCATCTTGCTCTTGC 3′ |
|  |  | Rv5′CGACGACACAGCCAGTAGAA 3′ |
|  | HvCOMT2 | Fw5′GTCCACCCATCTCATGCTCT 3′ |
|  |  | Rv5′AATCGACCGACGACACAAC 3′ |
|  | HvCOMT3 | Fw5′CCACATCGACCTCATTATGC 3′ |
|  |  | Rv5′GAATGGACCAATGGCACAAC 3′ |
|  | HvSnRK1 (HarvEST_U35_1211) | Fw5′ATGGGCTCTCGGTCTTCAGT 3′ |
|  |  | Rv5′AGGACTCCACCTGCACTTCA 3′ |
|  | HvRPII36  (HarvEST_U35_2804) | Fw5′GGAATTGGAAAGGACCATGC 3′ |
|  |  | Rv5′GCTCTCCACCCAGCTTTGTT 3′ |
| Generation of RNAi construct | HvCOMT1 | Fw5′**GGGGACAAGTTTGTACAAAAAAGCAGGCTTA**CATGGCGGCGCTCGCGCT 3’ |
|  |  | Rv5’**GGGGACCACTTTGTACAAGAAAGCTGGGTA**CTCCCTCTCGTACCTCTCCCTGCC 3’ |
| Generation of recombinant protein | HvCOMT1 | Fw5’**GGGGACAAGTTTGTACAAAAAAGCAGGCTTA**ATGGGCTCCACCGCAGCGGAC 3’ |
|  |  | Rv5’**GGGGACCACTTTGTACAAGAAAGCTGGGT**CTACTTGGTGAACTCGATGG3’ |

Table S 3. Summary of reaction set-up and cycling conditions for real-time PCR.

| reaction set-up | 10 μl 2X GoTaq qPCR Master Mix |
| --- | --- |
|  | 0.2 μl 100X ROX dye |
|  | 0.5 μM Fw and Rv primer |
|  | 1 μl of cDNA or water |
|  | 20 µl total volume |
| cycling conditions on StepOnePlus machine | 95°C for 10 min |
|  | 40 cycles of 95°C for 15 sec and 60°C for 1 min |
|  | followed by melt curve analysis |
|  | 95°C for 15 sec |
|  | 60°C for 1 min then increases of 0.3°C every 15 sec until 95°C |

Table S 4. The number of the conserved residues present for the binding/positioning of COMT substrates ferulic acid and 5-hydroxyconiferaldehyde. These residues were identified by Zubieta et al. (2002) in MsCOMT.

| Gene | Number of COMT substrate binding and positioning residues |
| --- | --- |
| MsCOMT | 13/13 |
| HvCOMT1(HORVU7Hr1G082280.1) | 12/13 |
| HvCOMT2(HORVU3Hr1G116770.1) | 12/13 |
| HvCOMT3(HORVU6Hr1G000040.1) | 12/13 |
| HvCOMTL1(HORVU1Hr1G011930.1) | 3/13 |
| HvCOMTL2(HORVU1Hr1G003370.2) | 3/13 |
| HvCOMTL3(HORVU7Hr1G119480.2) | 4/13 |
| HvCOMTL4(HORVU4Hr1G001250.1) | 4/13 |
| HvCOMTL5(HORVU1Hr1G089620.1) | 8/13 |
| HvCOMTL6(HvAK363914) | 5/13 |
| HvCOMTL7(HvAK373701) | 5/13 |
| BdCOMT(Bd3g16530.1) | 12/13 |
| BdCOMTL1(Bd2g02380.1) | 4/13 |
| BdCOMTL2(Bd2g02390.1) | 4/13 |
| BdCOMTL3(Bd1g14870.1) | 8/13 |
| Bd3g55890.1 | 4/13 |
|  |  |

Table S 5. Demonstration of the shared synteny between the barley and wheat chromosome arms that the barley *COMT* genes and homologous wheat gene(s) map to.

| barley *COMT* gene (the chromosome arm the gene maps to as identified in this study) | homologous wheat gene on phylogenetic tree in Figure 1 (chromosome arm the gene maps to and source of data) |
| --- | --- |
| *HvCOMT1*(HORVU7Hr1G082280.1) (7HL) | *TaOMT1_ AAP23942* (7BL) (Jung et al., 2008) |
| *HvCOMT2*(HORVU3Hr1G116770.1) (3HL) | *TaCM_ Q38J50.1* (3BL) (Ma, 2009)  *TaCOMT-3D* (3DL) (Wang et al., 2018) |
| *HvCOMT3*(HORVU6Hr1G000040.1) (6HS) | *TaOMT4_ EF423611* (6DS) (Jung et al., 2008)  *TaOMT5_ EF423610* (6BS) (Jung et al., 2008) |

Table S 6. Summary of the source tissue of ESTs for HvCOMT1, HvCOMT2 and HvCOMT3 from HarvEST #35.

|  | tissue | | | | |
| --- | --- | --- | --- | --- | --- |
| gene | leaf | root | rachis | other | total ESTs |
| *HvCOMT1* | 37 | 30 | 34 | 44 | 145 |
| *HvCOMT2* | 3 | 5 | 28 | 3 | 39 |
| *HvCOMT3* | 0 | 25 | 0 | 0 | 25 |

Table S 7. Summary of lignin data from the COMT RNAi lines. SE = Standard error. EFCW = extract free cell wall. For COMTRNAi_1, 5, 26 and 28, one of the three plants was a hemizygote.

| line | %KL (mean of duplicate measurements) | SE | (H+G+S) µmol/gr KL | SE | S/G | SE | % H/(H+G+S) | SE | % G/(H+G+S) | SE | % S/(H+G+S) | SE | 5-OHG (µmol/gr KL) | SE |
| --- | --- | --- | --- | --- | --- | --- | --- | --- | --- | --- | --- | --- | --- | --- |
| 1 | 17.13 | 1.6 | 1087.00 | 87.8 | 0.98 | 0.1 | 2.40 | 0.2 | 49.4 | 1.3 | 48.1 | 1.4 | 58.80 | 7.9 |
| 4 | 16.80 | 0.5 | 1298.33 | 106.6 | 0.79 | 0.0 | 2.70 | 0.2 | 54.3 | 1.0 | 43.0 | 1.2 | 37.87 | 7.9 |
| 4-azygote | 19.88 | 0.25 | 1514.67 | 42.58 | 1.74 | 0.10 | 2.43 | 0.17 | 35.70 | 1.25 | 61.90 | 1.30 | 2.20 | 0.21 |
| 5 | 18.35 | 0.8 | 1215.00 | 86.9 | 0.77 | 0.1 | 2.70 | 0.12 | 55.3 | 2.6 | 42.0 | 2.7 | 33.40 | 6.5 |
| 5-azygote | 18.57 | 0.17 | 1635.00 | 65.64 | 1.59 | 0.10 | 2.67 | 0.42 | 37.60 | 1.39 | 59.73 | 1.80 | 3.00 | 0.31 |
| 14 | 18.92 | 0.7 | 1206.67 | 36.7 | 0.80 | 0.0 | 2.40 | 0.06 | 54.2 | 1.0 | 43.4 | 1.0 | 55.17 | 5.9 |
| 14-azygote | 20.61 | 0.46 | 1642.00 | 9.54 | 1.67 | 0.04 | 2.00 | 0.15 | 36.77 | 0.44 | 61.23 | 0.56 | 2.73 | 0.13 |
| 19 | 16.62 | 0.45 | 1292.00 | 72.57 | 0.83 | 0.02 | 2.47 | 0.09 | 53.53 | 0.50 | 44.07 | 0.54 | 36.73 | 2.32 |
| 19-azygote | 17.93 | 0.37 | 1575.00 | 55.11 | 1.64 | 0.07 | 2.47 | 0.03 | 37.00 | 0.91 | 60.53 | 0.92 | 3.40 | 0.35 |
| 26 | 17.44 | 0.3 | 1189.67 | 48.1 | 0.67 | 0.0 | 2.47 | 0.19 | 58.2 | 0.6 | 39.3 | 0.4 | 23.80 | 6.61 |
| 26-azygote | 18.66 | 0.25 | 1423.00 | 72.45 | 1.48 | 0.10 | 2.73 | 0.15 | 39.40 | 1.45 | 57.87 | 1.59 | 1.97 | 0.09 |
| 28 | 18.57 | 0.6 | 1239.67 | 15.0 | 0.80 | 0.1 | 2.10 | 0.17 | 54.5 | 1.9 | 43.4 | 1.8 | 30.97 | 4.04 |
| 28-azygote | 20.64 | 0.66 | 1719.67 | 59.57 | 1.63 | 0.08 | 2.20 | 0.15 | 37.17 | 1.01 | 60.63 | 1.13 | 4.37 | 0.55 |
| wild type | 20.54 | 0.3 | 1679.33 | 46.7 | 1.64 | 0.1 | 2.57 | 0.20 | 36.9 | 1.2 | 60.5 | 1.4 | 3.10 | 0.17 |
| empty vector | 19.36 | 0.6 | 1771.00 | 42.6 | 1.59 | 0.0 | 2.63 | 0.15 | 37.6 | 0.7 | 59.8 | 0.8 | N/A | N/A |

Table S 8. m/z traces with a different intensity in the internodes of COMT RNAi lines as compared to empty vector and wild type controls. tR: retention time, Δppm: mass difference between m/z experimental and m/z theoretical in parts per million, s.e.m.: standard error of the mean, b.d.l.: below detection limit (set at 100 counts). (SEPARATE FILE .xls).

Table S 9. Microarray transcriptome analysis of internodes from two COMT RNAi lines (COMTRNAi_4 and COMTRNAi_14) and controls (empty vector and wild type). Genes that were significantly differentially expressed (*p* < 0.01) in both COMT RNAi lines compared to controls with at least a 3-fold change in expression are listed below in order of the magnitude of the fold change in expression.

| Primary Accession for microarray probe (corresponding barley genome identifier) | Control/  COMT Ratio | Gene annotation |
| --- | --- | --- |
|  |  |  |
| Upregulated genes in COMT RNAi lines | | |
| MLOC_22093.1(HORVU2Hr1G002840.4) | 0.18 | Lectin-like receptor protein kinase |
| MLOC_62697.1(no match) | 0.22 | Unknown function |
| MLOC_77305.1(no match) | 0.25 | F-box protein |
| AK365278(no match) | 0.28 | Methyl esterase; a/b hydrolase family |
|  |  |  |
| Downregulated genes in COMT RNAi lines | | |
| MLOC_65378.1(HORVU7Hr1G082280.1) | 24.78 | HvCOMT1 |
| MLOC_67735.1(HORVU6Hr1G010420.3) | 6.57 | Proteasome regulator (PTRE1) |
| AK368196(HORVU2Hr1G017420.2) | 6.44 | Lectin, carbohydrate binding |
| MLOC_31106.1(no match) | 6.01 | Cyclin B1 |
| AK364509(HORVU3Hr1G042440.23) | 5.27 | NRAMP metal transporter |
| MLOC_51403.1(no match) | 5.00 | Kinase regulator, membrane associated |
| AK376048(HORVU7Hr1G093190.7) | 4.57 | Galactan synthase |
| AK365088 (HORVU6Hr1G092840.2) | 3.92 | O-methyltransferase |
| MLOC_65231.1(HORVU3Hr1G116220.1) | 3.89 | Dof-type Zn finger transcription factor, salicylic acid response |
| AK251685.1(HORVU3Hr1G099590.2) | 3.83 | Cysteine-rich RLK protein, DUF26 |
| MLOC_25573.2(no match) | 3.6 | DnaJ heat shock protein, chloroplast development |
| MLOC_77726.1(no match) | 3.49 | Unknown function, embryo development |
| AK364095(no match) | 3.11 | Tubby-like protein/F-box protein (TLP) |
| MLOC_23912.1(HORVU6Hr1G078350.1) | 3.01 | NF-X1 like zinc finger transcription factor |

**Method S1**

## Sequence retrieval, data sources and multiple alignment

Barley, brachypodium and rice sequences with >40% identity to maize *ZmCOMT* (M73235) were retrieved by BLASTp or tBLASTn searches. Databases searched for sequences with >40% identity to ZmCOMT were: brachypodium protein database v.1 at MIPS <http://mips.helmholtz-muenchen.de/plant/brachypodium/searchjsp/index.jsp>; MSU rice genome annotation project release 7 at <http://rice.plantbiology.msu.edu/analyses_search_blast.shtml>; barley databases HarvEST#35 at <http://harvest-web.org/hweb/mainmenu.wc>, barley pseudomolecule genome assembly (Mascher *et al.*, 2017) and Morex BAC contigs at <http://webblast.ipk-gatersleben.de/barley/viroblast.php> and several in-house databases. This threshold captured rice genes previously annotated as *COMT-likes* (Hamberger, (2007) and similar barley and brachypodium genes (also described here as *COMT-likes*). For phylogenetic analysis we also included published the COMT genes listed in the main paper. Full length protein sequences were aligned by MUSCLE (Edgar, 2004) implemented in MEGA7 (Kumar *et al.*, 2016) with default parameters. Gblocks (Castresana, 2000) <http://molevol.cmima.csic.es/castresana/Gblocks_server.html> removed poorly aligned sections.

**Method S2**

**Determination of the genomic location and evidence for expression of barley genes**

The genomic location of the barley genes was determined from two sources where possible. Firstly, mapping data from microarray experiments from wheat-barley chromosome addition lines (Bilgic et al., 2007) were queried with the Barley1 GeneChip probesets for *HvCOMT* and *HvCOMT-like* genes listed in Table S1. Secondly, NGS sequence of barley cv. Betzes generated from flow sorted chromosome arms was queried at http://webblast.ipk-gatersleben.de/barley/viroblast.php (Database: SortedChromosomes) to identify which chromosome arm *HvCOMT* and *HvCOMT-like* genes were a match with. The presence of a matching HarvEST35 unigene or ESTs in GenBank was considered evidence for expression of a barley gene. When no ESTs were identified, a database of NGS sequence from JHI referred to here as Barley Transcriptome database (restricted access) was also searched.

**Method S3**

**Generation and purification of a recombinant HvCOMT1 protein for antibody production**

Primers containing Gateway AttB sites (Table S5) were used to amplify the HvCOMT1 ORF from cv. Optic cDNA with the Phusion proofreading polymerase (Finnzymes, Finland). The PCR product was cloned into the pGEMT-easy vector (Promega) and sequenced. The pGEMT-HvCOMT1ORF construct was recombined with the pDONR201 vector in a BP reaction. The pDONR-HvCOMT1ORF construct was then recombined in an LR reaction with the N–terminal GST tagged expression vector pGEX-5G/LIC (*tac* IPTG inducible promoter) (GE Life Sciences, UK). This vector has previously been modified to facilitate Gateway cloning. BP and LR reactions were performed as described in previous section. The pGEX-HvCOMT1ORF construct was used to transform the *Escherichia coli* strain BL21 (DE3) (Novagen, USA) by electroporation. The transformed BL21 (DE3) line was cultured in NZCYM medium with 100 μg·ml^-1^ ampicillin at 37°C to an OD_600nm_ of 0.6 and then 1 mM IPTG was used to induce the over-expression of the recombinant protein followed by incubation O/N at 18°C. The cells were harvested by centrifugation and lysed with Bugbuster Master Mix (Novogen) supplemented with Complete (Roche) protease inhibitors. The GST-tagged COMT protein was purified from the clarified lysate by affinity chromatography using glutathione agarose resin (Sigma) according to the manufacturer’s instructions. The COMT protein was cleaved from the GST tag on the resin with thrombin protease (Amersham Biosciences, UK) (50 units) O/N at room temperature followed by elution of the COMT protein from the resin. The GST tag was also eluted from the resin with reduced glutathione (Sigma) to determine the efficiency of cleavage. Quantification and SDS-PAGE of the proteins were performed as described in previous section. The recombinant protein was diluted to aliquots of 300 μg·ml^-1^ in 2M urea for immunisation of rabbits by Dundee Cell Products Ltd, Dundee, UK.

**Method S4**

**Southern Blot**

gDNA was extracted from young leaves of T0 COMT RNAi lines with the DNeasy Plant Maxi Kit (Qiagen, UK) according to the manufacturer’s instructions. Between 5-10 µg of gDNA was digested separately with the restriction endonucleases HindIII or EcoRV according to standard protocols. HindIII and EcoRV each digest once within the T-DNA fragment from the pIPKb007-HvCOMT1-RNAi construct. The digested gDNA was separated in a 0.8% w/v agarose gel overnight at 30V. The gel was depurinated, denatured, neutralised and then blotted onto an Amersham Hybond -N^+^ (GE Heathcare, UK) membrane by capillary transfer under neutral conditions as described in the ‘DIG Application Manual for Filter Hybridisation (2008)’, (Roche, UK). The membrane was crosslinked with UV light using the Optimal Crosslink setting on the Spectrolinker XL-1500 UV crosslinker (Spectronics Corporation, USA). The pre-hybridisation (68°C), hybridisation (68°C) and wash steps (65°C) were preformed as described by Engler-Blum et al. (1993). A 707 bp probe for the *hpt* gene was labelled with the non-isotopic DIG (digoxigenin)-11-dUTP label (Roche) by PCR as follows: the GoTaq polymerase (Promega, UK) was used with the cycling conditions of 95°C for 2 min, followed by 35 cycles of 94°C for 30 sec, 52°C for 45 sec, 72°C for 1 min and 72°C for 10 min with the *hpt* probe Fw 5′ GATCGGACGATTGCGTCGCA 3′ and Rv 5′ TATCGGCACTTTGCATCGGC 3′ primers at a 1μM final concentration. For template of the *hpt* gene, 10 ρg of the pIPKb007 vector was used. In this labelling PCR reaction, the nucleotides dATP, dGTP and dCTP were at a 200 μM final concentrations and dTTP and DIG-11-dUTP at 165 μM and 35 μM final concentrations respectively. For probing of the membrane, 1 μl of the labelled PCR product (probe) was added to the hybridisation solution for every 2.5 cm^2^ of membrane. The probe was detected with the anti-DIG-AP conjugate antibody (Roche) and visualised with the CDP-star (GE Healthcare) chemiluminescence substrate by exposure to Amersham Hyperfilm ECL (GE Heathcare) generally as described by Engler-Blum et al. (1993).

**Method S5**

**RNA extraction and microarray processing for transcriptome analysis**

RNA was isolated from 100 mg of ground stem material for each of three replicates for each transgenic line using the Qiagen RNeasy Plant Mini kit with on column DNase treatment. RNA quality was confirmed using an Agilent Bioanalyzer (RNA integrity number ≥ 8 for all samples). 670 ng RNA for each sample was submitted to the James Hutton Institute Functional Genomics facility, where RNA was labelled as cDNA and hybridized against a custom barley 60K Agilent microarray chip derived from predicted transcripts of the barley reference sequence assembly (IBSC, 2012) and full-length cDNAs. Chips were scanned using an Agilent G2565B scanner and data extracted using Agilent FE software. Probes that were not detectable in at least two samples were not included in analyses.

**Method S6**

**Phenolic metabolome analysis**

For metabolite analysis, 100 mg ground stem was extracted using 1 mL HPLC grade methanol (Fisher Scientific, UK) at 70^o^C for 15 min. Supernatants were dried in a vacuum centrifuge. Pellets were dissolved in 100 µL cyclohexane followed by 100 µL water. The tubes were vortexed and centrifuged at 14,000 rpm (20,000 x g) for 10 min; 80ul of the water phase was transferred to a 96 well plate. For LC-MS analysis, 15 µL of the water phase was injected on an Ultra High Performance Liquid Chromatography (UHPLC) system (Waters Acquity UPLC®) equipped with a BEH C18 column (2.1 x 150 mm, 1.7 µM, Waters) coupled to a time-of-flight mass spectrometer (TOF MS, Synapt Q-Tof (Waters Corporation, Milford, Massachusetts, USA)). For chromatographic separation, a gradient of two buffers was used: buffer A (99/1/0.1 H_2_O/ACN/formic acid pH3), buffer B (99/1/0.1 ACN/H_2_O/formic acid pH3); 95% A for 0.1 min decreased to 50% A in 30 min (350 µL/min, column temperature 40 °C). The flow was diverted to the mass spectrometer equipped with an electrospray ionization source operating in negative mode with the lockspray interface enabled for accurate mass measurements. The MS source parameters were: capillary voltage, 2.5 kV; sampling cone, 37 V; extraction cone, 3.5 V; source temperature, 120 °C; desolvation temperature, 400 °C; cone gas flow, 50 L/h; and desolvation gas flow, 550 L/h. The collision energy for the trap and transfer cells was 6 V and 4 V, respectively. For data acquisition, the dynamic range enhancement mode was activated. Full-scan data were recorded in centroid V-mode; the mass range was set between *m/z* 100 and 1,200, with a scan speed of 0.2 s/scan, data was acquired through Masslynx software (Waters). Leucin-enkephalin (250 pg/μL solubilized in water/ACN 1:1 (vol:vol), with 0.1% formic acid) was used for the lock mass calibration, scanning every 10 s for 0.5 s. For MS/MS purposes, the collision energy in the trap cell was ramped from 10 to 20 eV for Low Mass and from 20 to 45 eV for High Mass. Data processing was done with Progenesis QI software (Waters). For identification, compound ions that contained fragmentation data were matched against an in-house library (PhytoComp) and accepted based on their score for retention time match, fragmentation pattern similarity and mass accuracy tolerance.

References

Bilgic, H., Cho, S., Garvin, D.F. and Muehlbauer, G.J. (2007) Mapping barley genes to chromosome arms by transcript profiling of wheat-barley ditelosomic chromosome addition lines. *Genome* **50**, 898-906.

Castresana J. 2000. Selection of conserved blocks from multiple alignments for their use in phylogenetic analysis. *Molecular Biology and Evolution* **17**(4): 540-552.

Edgar RC. 2004. MUSCLE: multiple sequence alignment with high accuracy and high throughput. *Nucleic Acids Research* **32**(5): 1792-1797.

Engler-Blum, G., Meier, M., Frank, J. and Muller, G.A. (1993) Reduction of background problems in nonradioactive northern and Southern blot analyses enables higher sensitivity than 32P-based hybridizations. *Anal. Biochem.* **210**, 235-244.

Hamberger B, Ellis M, Friedmann M, Souza CDA, Barbazuk B, Douglas CJ. 2007**.** Genome-wide analyses of phenylpropanoid-related genes in *Populus trichocarpa*, *Arabidopsis thaliana*, and *Oryza sativa*: the *Populus* lignin toolbox and conservation and diversification of angiosperm gene families. *Canadian Journal of Botany* **85**(12): 1182-1201.

Ho-Yue-Kuang S, Alvarado C, Antelme S, Bouchet B, Cézard L, Le Bris P, Legée F, Maia-Grondard A, Yoshinaga A, Saulnier L, et al. (2016). Mutation in Brachypodium caffeic acid O-methyltransferase 6 alters stem and grain lignins and improves straw saccharification without deteriorating grain quality. *Journal of Experimental Botany* **67**(1): 227-237.

IBSC (2012) A physical, genetic and functional sequence assembly of the barley genome. *Nature* **491**, 711–716.

Jung, J., Hong, M., Kim, D., Kim, J., Heo, H., Kim, T., Jang, C. and Seo, Y. (2008) Structural and expressional divergence of genes encoding O-methyltransferase in wheat. *Genome* **51**, 856-869.

Kumar S, Stecher G, and Tamura K. 2016. MEGA7: Molecular evolutionary genetics analysis version 7.0 for bigger datasets. *Molecular Biology and Evolution* **33**: 1870-1874.

Ma, Q.-H. (2009) The expression of caffeic acid 3-O-methyltransferase in two wheat genotypes differing in lodging resistance. *J. Exp. Bot.* **60**, 2763-2771.

Mascher, M., Gundlach, H., Himmelbach, A., Beier, S., Twardziok, S.O., Wicker, T., Radchuk, V., Dockter, C., Hedley, P.E., Russell, J., Bayer, M., Ramsay, L., Liu, H., Haberer, G., Zhang, X.-Q., Zhang, Q., Barrero, R.A., Li, L., Taudien, S., Groth, M., Felder, M., Hastie, A., Šimková, H., Staňková, H., Vrána, J., Chan, S., Muñoz-Amatriaín, M., Ounit, R., Wanamaker, S., Bolser, D., Colmsee, C., Schmutzer, T., Aliyeva-Schnorr, L., Grasso, S., Tanskanen, J., Chailyan, A., Sampath, D., Heavens, D., Clissold, L., Cao, S., Chapman, B., Dai, F., Han, Y., Li, H., Li, X., Lin, C., McCooke, J.K., Tan, C., Wang, P., Wang, S., Yin, S., Zhou, G., Poland, J.A., Bellgard, M.I., Borisjuk, L., Houben, A., Doležel, J., Ayling, S., Lonardi, S., Kersey, P., Langridge, P., Muehlbauer, G.J., Clark, M.D., Caccamo, M., Schulman, A.H., Mayer, K.F.X., Platzer, M., Close, T.J., Scholz, U., Hansson, M., Zhang, G., Braumann, I., Spannagl, M., Li, C., Waugh, R. and Stein, N. (2017) A chromosome conformation capture ordered sequence of the barley genome. *Nature* **544**, 427-433.

Morreel, K., Dima, O., Kim, H., Lu, F., Niculaes, C., Vanholme, R., Dauwe, R., Goeminne, G., Inze, D., Messens, E., Ralph, J. and Boerjan, W. (2010a) Mass spectrometry-based sequencing of lignin oligomers. *Plant Physiol.* **153**, 1464-1478.

Morreel, K., Kim, H., Lu, F., Dima, O., Akiyama, T., Vanholme, R., Niculaes, C., Goeminne, G., Inzé, D., Messens, E., Ralph, J. and Boerjan, W. (2010b) Mass Spectrometry-Based Fragmentation as an Identification Tool in Lignomics. *Anal. Chem.* **82**, 8095-8105.

Tsuji, Y., Vanholme, R., Tobimatsu, Y., Ishikawa, Y., Foster, C.E., Kamimura, N., Hishiyama, S., Hashimoto, S., Shino, A., Hara, H., Sato, I.K., Oyarce, P., Goeminne , G., Morreel, K., Kikuchi, J., Takano, T., Fukuda, M., Katayama, Y., Boerjan, W., Ralph, J., Masai, E. and Kajita, S. (2015) Introduction of chemically labile substructures into Arabidopsis lignin through the use of LigD, the Cα‐dehydrogenase from Sphingobium sp. strain SYK‐6. *Plant Biotechnol. J.* **13**, 821-832.

Wang, M., Zhu, X., Wang, K., Lu, C., Luo, M., Shan, T. and Zhang, Z. (2018) A wheat caffeic acid 3-O-methyltransferase TaCOMT-3D positively contributes to both resistance to sharp eyespot disease and stem mechanical strength. *Sci Rep* **8**, 6543.

Zubieta, C., Kota, P., Ferrer, J.-L., Dixon, R.A. and Noel, J.P. (2002) Structural basis for the modulation of lignin monomer methylation by caffeic acid/5-hydroxyferulic acid 3/5-O-methyltransferase. *Plant Cell* **14**, 1265-1277.
